# Economic Evaluations of Pharmacogenetic and Pharmacogenomic Screening Tests: A Systematic Review. Second Update of the Literature

**DOI:** 10.1371/journal.pone.0146262

**Published:** 2016-01-11

**Authors:** Elizabeth J. J. Berm, Margot de Looff, Bob Wilffert, Cornelis Boersma, Lieven Annemans, Stefan Vegter, Job F. M. van Boven, Maarten J. Postma

**Affiliations:** 1 Unit of Pharmacotherapy & Pharmaceutical Care, Department of Pharmacy, University of Groningen, Groningen, The Netherlands; 2 Unit of PharmacoEpidemiology & PharmacoEconomics, Department of Pharmacy, University of Groningen, Groningen, The Netherlands; 3 GSK, Zeist, The Netherlands; 4 Department of Clinical Pharmacy and Pharmacology, University Medical Center Groningen, University of Groningen, Groningen, The Netherlands; 5 Faculty of Medicine & Health Science, Department of Public Health, Ghent University, Ghent, Belgium; 6 Institute of Science in Healthy Ageing & health care (SHARE), University Medical Center Groningen, University of Groningen, Groningen, The Netherlands; University Hospital Oldenburg, GERMANY

## Abstract

**Objective:**

Due to extended application of pharmacogenetic and pharmacogenomic screening (PGx) tests it is important to assess whether they provide good value for money. This review provides an update of the literature.

**Methods:**

A literature search was performed in PubMed and papers published between August 2010 and September 2014, investigating the cost-effectiveness of PGx screening tests, were included. Papers from 2000 until July 2010 were included via two previous systematic reviews. Studies’ overall quality was assessed with the Quality of Health Economic Studies (QHES) instrument.

**Results:**

We found 38 studies, which combined with the previous 42 studies resulted in a total of 80 included studies. An average QHES score of 76 was found. Since 2010, more studies were funded by pharmaceutical companies. Most recent studies performed cost-utility analysis, univariate and probabilistic sensitivity analyses, and discussed limitations of their economic evaluations. Most studies indicated favorable cost-effectiveness. Majority of evaluations did not provide information regarding the intrinsic value of the PGx test. There were considerable differences in the costs for PGx testing. Reporting of the direction and magnitude of bias on the cost-effectiveness estimates as well as motivation for the chosen economic model and perspective were frequently missing.

**Conclusions:**

Application of PGx tests was mostly found to be a cost-effective or cost-saving strategy. We found that only the minority of recent pharmacoeconomic evaluations assessed the intrinsic value of the PGx tests. There was an increase in the number of studies and in the reporting of quality associated characteristics. To improve future evaluations, scenario analysis including a broad range of PGx tests costs and equal costs of comparator drugs to assess the intrinsic value of the PGx tests, are recommended. In addition, robust clinical evidence regarding PGx tests’ efficacy remains of utmost importance.

## Introduction

Pharmacogenetics and pharmacogenomics investigate the influence of genetic and genomic variations on drug response in individuals [1]. The term pharmacogenetics covers the study of single genes, whereas pharmacogenomics is used to describe the study of several genes [1]. The abbreviation PGx is used to cover both pharmacogenetics and pharmacogenomics. PGx tests offer great opportunities for personalised medicine, by combining genetic information and corresponding phenotypes [2]. Ideally, by applying PGx, the most optimal, tailored pharmacotherapy can be determined, thereby increasing the treatment’s overall efficacy and decreasing the incidence of adverse events [1]. In the field of oncology it has been shown that for certain therapies the specific genetic variations in cancer cells can affect the drug efficacy and/or adverse events [2]. Hence, patients may benefit from the PGx tests by utilising an alternative therapy, or changing the drug dosage [3,4]. Therefore, PGx is nowadays often used as a synonym for personalised medicine, although personalized medicine is a much broader concept [2].

It is likely that an increasing amount of patient-specific genomic information will be available in the near future and this may result in an increased usage of PGx tests which needs evaluation of effects, but also cost effectiveness [5]. PGx has the potential to reduce the costs associated with inappropriate, expensive drug treatments and/or serious adverse drug reactions, in particular those that require hospitalisation [3]. Therefore, next to optimising health outcomes, PGx tests might be cost-saving [1,5]. However, in order to obtain valid, accurate, and relevant estimates of cost-effectiveness, reliable economic studies are required.

Economic evaluations of PGx tests entail some specific difficulties. Often there is no hard clinical evidence regarding the effects of the PGx test on the clinical utility and it is unlikely that such evidence will be available for the use of every genetic variant [6]. Furthermore, for PGx tests, compliance and adherence of clinicians to the test results might have an effect on the effectiveness of PGx tests which is hard to incorporate in a cost-effectiveness analysis [7]. Differences in costs for the PGx test can be substantial between countries, or even laboratories, and therefore it is advised to include different costs in scenario analysis [7]. In addition, the sensitivity and specificity of a PGx test can vary due to different ethnicities studied, or genetic variations analysed.

In the last decade, several reviews investigated economic evaluations of genetic tests [1,8–14]. These reviews showed that the level of consistency and quality could be improved. Many original studies lacked a thorough sensitivity analysis and moreover, in general a poor quality of methodology was noticed [9,11]. Inconsistencies mainly resulted from e.g. lack of clinical evidence, different methodologies as well as statistics and modest heterogeneity among study designs and patient populations [3,9]. However, these different methodologies have not been in detail dealt with in previous systematic reviews. Recent developments have led to a bifurcation in the nature of the economic evaluations of PGx testing in, on the one hand, studies assessing the intrinsic value of a test and, on the other hand, studies assessing the value of the test in combination with an active compound. For example in colorectal cancer, the economic value of testing for *KRAS* as compared to no testing could be considered the “intrinsic value” of the PGx test. *KRAS* testing before treatment with cetuximab, is found to be dominant (i.e. cost-saving and better) as compared to no prior testing and therefore it is recommended before administration of cetuximab [15]. By its uptake in clinical guidelines, a shift in the comparator for future economic evaluations took place, as the combination of cetuximab and *KRAS* testing became usual care. In future evaluations, the intrinsic value of the *KRAS* test itself will no longer be assessed, but rather the combination of a drug and its test as compared with a new treatment option. Following this development, a distinction between the nature of economic evaluations of PGx tests is important for a fair comparison of studies.

The objective of this study was to give recommendations for improvement and an update of the literature about PGx tests, taking into account the difference between the intrinsic value of tests themselves and tests embedded into economic evaluations as usual care or best current care. Our new findings were placed in perspective with respect to findings from our previous reviews [1,9]. As such, our study links together a period from 2000- September 2014 of PGx testing and pharmacoeconomics.

## Methods

A search in PubMed was performed using combinations of the following terms [PubMed search: all fields] and their thesauri variants: [‘cost-effectiveness [including MeSH)’ OR ‘cost-utility’ OR ‘cost-benefit’ OR ‘cost-minimization’ OR ‘pharmacoeconomics [including MeSH]’] AND [‘pharmaco-genetics’ OR ‘pharmacogenomics [including MeSH]’ OR ‘genotyping’ OR ‘genetic screening’ OR ‘genetic testing [including MeSH]’ OR ‘genotyped’ OR ‘polymorphism screening’]. These search terms were in line with the terms that were used in previous reviews, performed in 2008 and 2010 [1,9]. The search was last updated in October 2014 and studies were included if they were: published between August 2010 and September 2014, peer reviewed, performed on a genetic screening method of the human genome, evaluating economic outcomes, written in English, and the genetic or genomic variations were shown to influence the drug efficacy or drug safety. Articles were first screened on title. If the title was not informative enough to form a decision with respect to these criteria, abstracts were assessed. Additional articles were identified through reference tracking.

From the selected studies, the following data were extracted: (I) area of disease or patient population, (II) gene(s) analysed by the pharmacogenetic test, (III) the costs of the pharmacogenetic test, (IV) pharmaceutical compound influenced by the genetic variation, (V) type of economic analysis, (VI) type of sensitivity analysis, (VII) time horizon, (VIII) discounting, (IX) perspective, (X) the outcome measurements, and (XI) the funding body. For interpretation of the outcome measure (i.e. cost-effectiveness), the conclusions as reported by the authors were used. Furthermore, an assessment of the papers ‘discussion on the study limitations’ was made. In this assessment, all limitations mentioned by the authors were captured to look for common and uncommon themes. As stated in the previous review, assessment of these points is expected to provide good information for an adequate interpretation of the studies design, reporting, robustness, methodologies used, and statistical analyses performed [9]. In addition to these points, which were assessed in our previous reviews, we added (XII) reporting of analytical validity of the PGx test, (XIII) the cost-effectiveness threshold, (XIV) the country which was used for the perspective of the economic evaluation, and (XV) a weighted quality assessment for the studies included by this update. To assess the quality, the Quality of Health Economic Studies (QHES) instrument was used [16]. This instrument was used to improve generalisability of the results with respect to other reviews performed in the same field [10,13]. According to the QHES checklist a score between 0 and 100 was generated. A score of ≥ 75 was considered as a high quality score [11]. Two reviewers assessed the quality of the included studies, if results were different, consensus was reached through discussion.

Data from before 2008, and for the period 2008—July 2010 were retrieved from the two previous reviews by Vegter *et al*. (2008, 2010) [1,9].

## Results

### Included studies

**Fig 1:**
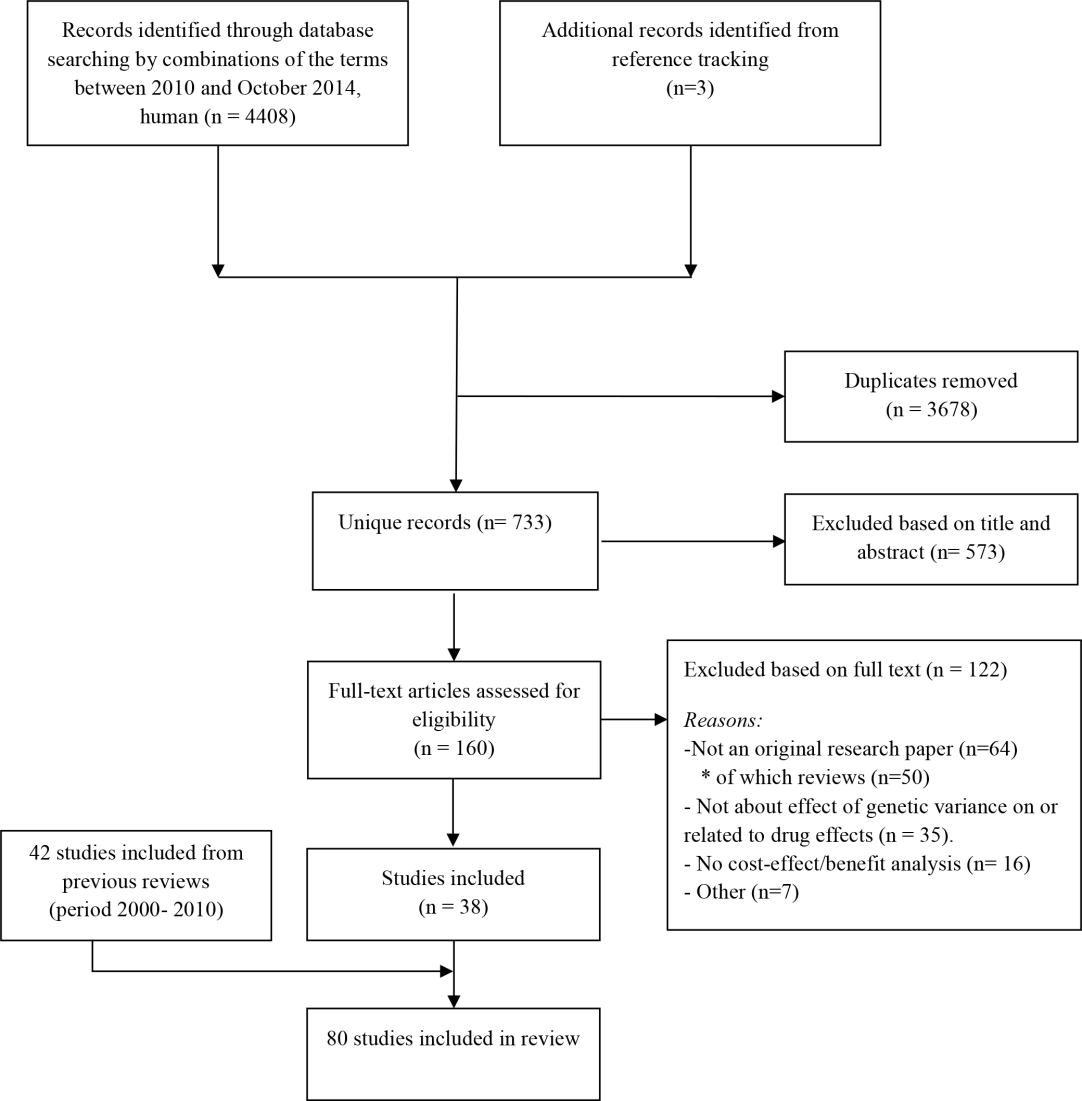
PRISMA flow chart of the literature search.

**Fig 2:**
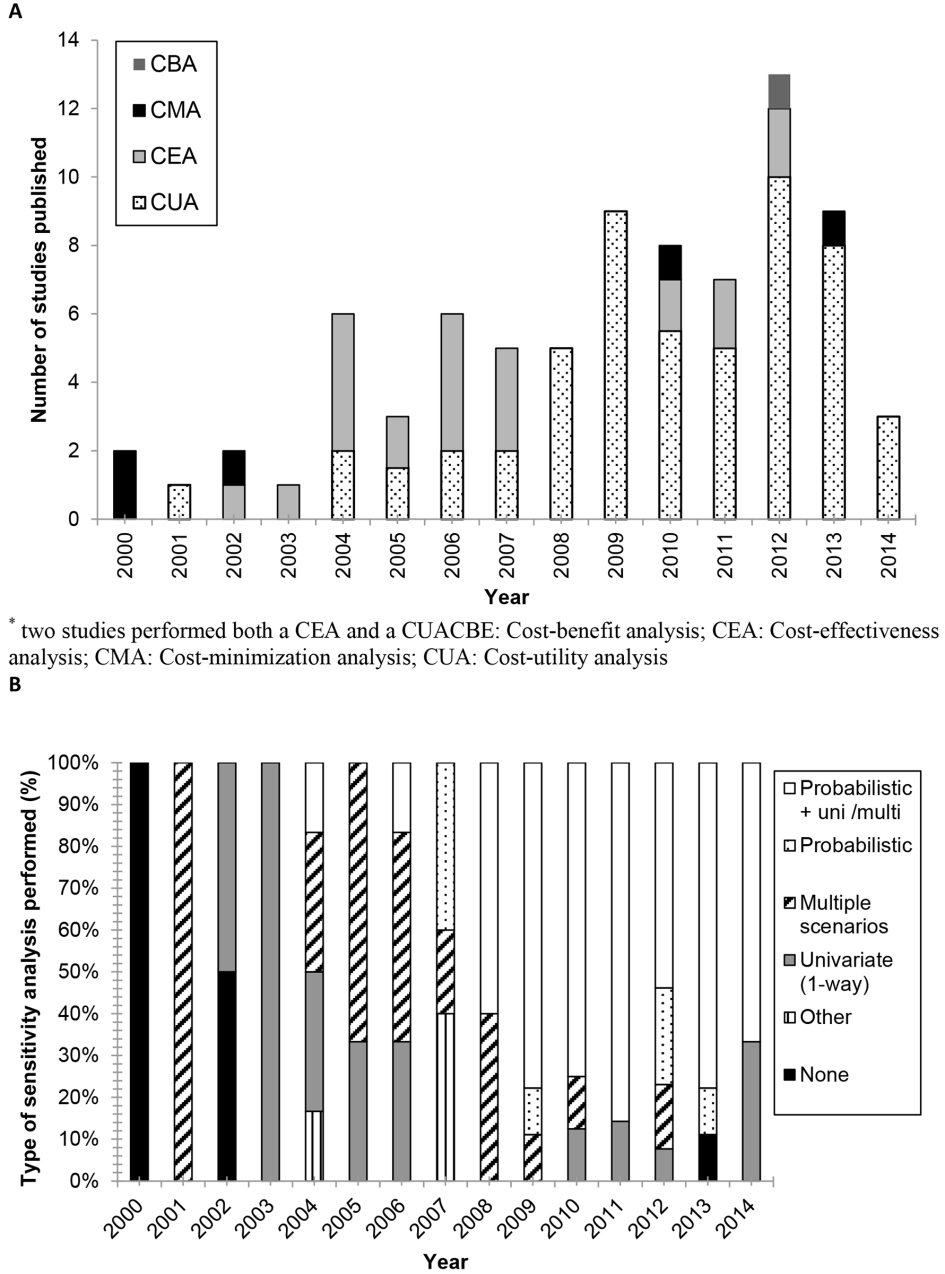
Type of outcome analysis (A) and sensitivity analysis (B) of PGx studies from 2000- September 2014.

Results of the search strategy are provided in a PRISMA flow chart (Fig 1) [17]. The PubMed search yielded 4408 hits. Duplicates were removed and out of the remaining 733 articles, 160 were selected for full text assessment. Three articles were identified through reference tracking. After inspection of full texts, 122 studies were excluded, resulting in 38 included studies [15,18–54]. Main reasons for exclusion were the type of genetic tests studied (i.e. not related to pharmacogenetics) and review papers. Subsequently, 42 studies published before July 2010 were added based on the two reviews by Vegter *et al*. [1,9,55–96], resulting in a final inclusion of 80 studies. Fig 2A provides an overview of the total of 80 studies, published from 2000 until September 2014, sorted by the type of pharmacoeconomic analysis performed. Most studies were published in 2012 (16%), with a total of 13 publications.

### Type of analysis: intrinsic value or combination with new drug

Cost utility analysis (CUA) was the technique mostly applied, namely in 54 studies (68%). Cost-effectiveness analysis (CEA) was performed in 20 studies (25%), cost minimization analysis (CMA) was used 5 times (6%). Note that for the sake of clarity we explicitly differentiate between CUA (with results expressed in cost per QALY) and CEA (with other parameters for effectiveness). Before 2008, CEA was the most frequently applied study type. Since 2008, CUA was performed in most of the publications. Some studies directly assessed the intrinsic value of the PGx test [15,18–22,24–28], while other studies applied scenarios in which equal costs and efficacy were assumed for the drug related to the PGx tests and the alternative treatment [23,29]. Both were considered cost-effectiveness estimates that provide an indication of the intrinsic value of the PGx test (Table 1). However, we found that the majority of the newly included (i.e. since 2010) CEAs incorporated a PGx test strategy in combination with a drug and compared this combination to another drug (Table 2). As such, the intrinsic value of the PGx test itself was not assessed. For example, Handorf *et al*. compared therapy for non-small cell lung cancer with a platinum combination to PGx selected treatment with erlotinib [44]. In this analysis, the cost-effectiveness of the PGx test itself was not assessed, but the combination of the PGx test and erlotinib. This makes the outcome mainly dependent on the price of erlotinib. The incremental cost-effectiveness ratios (ICER) of base case scenarios are shown in Tables 1 and 2.

**Table 1:** Outcomes and funding sources of pharmaco-economic PGx studies on the intrinsic value of PGx test. Published between August 2010 and September 2014. Note that numbers were rounded towards hundreds.

**1**^**st**^ **Author (reference)**	**PGx test**	**Analytical validity PGx test reported**	**OutcomeMeasure**	**Quantitative outcome or ICER (US$)**	**Cost effectiveness threshold ($/QALY)**	**Conclusion based on outcome**	**Funding**	**Country**	**QHES score**
**Klang 2010 [18]**	*21 gene assay*	No	QALY	$10,800	No number	Cost-effective	Teva Pharmaceutical Industries Ltd.	Israel	83
**Bacchi 2010 [19]**	*21- gene assay*	No	Costs	$800 saved per patient	n.a.	Cost-saving	Unknown	Brazil	34
**Hall 2012 [20]**	*21 gene assay*	No	QALY	$8,900	£20,00–30,00 ($31,200–46,700)	Cost-effective, though substantial uncertainty	Academic resources	UK	90
**Vanderlaan2011 [21]**	*21 gene assay*	No	QALY	$4,4009 saved per patient per year	n.a.	Dominant	Genomic Health, Inc.	USA	31
**Verhoef 2013 [22]**	*CYP2C9* and *VKORC1*	No	QALY	€2,700 ($3200)	€20,000 ($24,200)	Cost-effective	European grant	Netherlands	87
**Dong 2012 [23]**	*HLA-B* *1502	Yes	QALY	$29,800*	$50,000	Cost-effective for Singaporean Chinese and Malays, but not for Singaporean Indians.	Academic resources	Singapore	63
**Tiamkao 2013 [24]**	*HLA-B* *1502	Yes	Costs	98,600 baht ($3,000) saved per 100 cases	n.a.	Cost-effective	None	Thailand	48
**Shiroiwa 2010 [15]**	*KRAS*	No	QALY and LYG	Dominant	n.a.	Dominant	Roche Diagnostics K.K.	Japan	90
**Blank 2011 [25]**	*KRAS* and *BRAF*	Yes	QALY	KRAS and BRAF saves €3,300 ($2,500) per patient	n.a.	Cost saving	Academic resources	Switzerland	83
**Behl 2012 [26]**	*KRAS* and/or *BRAF*	No	LYS	KRAS alone saves $7,500 per patient	n.a.	Cost saving	Academic resources	USA	84
				Additional BRAF testing saves $1,000 per patient					
**Shiffman 2012 [27]**	*LPA*	No	QALY	$25,000	No number	Could be cost-effective	Berkeley HeartLab, Inc.	USA	34
**Donnan 2011 [28]**	*TPMT*	Yes	Life months	Cost no test CAN$700 ($600) per patient- Cost genetic test CAN$1,100 ($900) per patients	n.a.	Not cost-effective	Academic resources	Canada	77
				With test no LY gained					
**Schackman 2013 [29]**	*UGT1A1*	Yes	QALY	$2,000,000*	$100,000	Not cost-effective unless assay cost are low	Academic resources	USA	83

*Authors assumed equal costs and efficacy for different pharmaceuticals

BRAF, v-Raf murine sarcoma viral oncogene homolog B1; HLA, human leukocyte antigen; HSR, hyper sensitivity reaction; ICER, incremental cost-effectiveness ratio; KRAS, Kirsten rat sarcoma viral oncogene homolog; LPA, lipoprotein-a; LYG, life-years-gained; LYS, life-years-saved; PGx, pharmacogenetic; QALY, quality-adjusted-life-year; TPMT, thiopurine S-methyltransferase; UGT, UDP-glucuronosyltransferase.

**Table 2:** Outcomes and funding sources of pharmaco-economic PGx studies (of treatment comparisons involving PGx testing. Published between August 2010 and September 2014. Note that numbers were rounded towards hundreds. Italic quantitative outcomes (ICERs) are not related to PGx scenario.

1^st^ Author (reference)	PGx test	Analytical validity PGx test reported (y/n)	Outcom Measure	Quantitative outcome or ICER (US$)	Cost-effectiveness threshold ($/QALY)	Conclusion based on outcome	Funding	Country	QHES score
**Olgiati** 2012 [30]	*5-HTTLPR*	No	QALW	Euro A $1,100	< 3 times the GDP per capita	Cost-effective in high-income countries	Unknown	Europe	61
				Euro B $1,200					
				Euro C $1,200					
**Serretti** 2011 [31]	*5-HTTLPR*	No	QALW	$2,900	$50,000	Not cost-effective	Unknown	Italy	87
**Reed** 2011 [32]	8–14 risk alleles	No	QALY	$98,100	$100,000	Cost-effective	National Cancer Institute	USA	83
				$103,200					
**Djalalov** 2012 [33]	*APOE ε4*	yes	QALY	CAN$38,000 ($32,700)	No number	May be economically attractive	Academic resources	Canada	90
**Kazi** 2014 [34]	*CYP2C19*	No	QALY	*Extendedly dominated*	$50,000	may improve cost effectiveness	American Heart Association and academic resources	USA	90
				*$52*,*600*					
				$35,800					
				$30,200					
**Reese** 2012 [35]	*CYP2C19*	No	CVE avoided	Cost saving	No number	Dominant	Unknown	USA	64
				Cost saving					
				$2,300					
				Cost saving					
**Sorich** 2013 [36]	*CYP2C19*	No	QALY	AUS$6,300 ($5,200)	AUS$30,00–50,000 ($24,500–40,800)	Cost-effective	Heart Foundation of Australia	Australia	75
				*AUS$22*,*800 ($18*,*600)*					
**Crespin** 2011 [37]	CYP2C19 *2	Yes	QALY	$10,100	$50,000	Cost-effective	Academic resources	USA	93
**Lala** 2013 [38]	CYP2C19 *2	Yes	QALY	Dominant	n.a.	Dominant	Academic resources	USA	83
				Dominant					
**Panattoni** 2012 [39]	CYP2C19 *2	No	QALY	NZ$ 24,600 ($19,200)	NZ$50,000 ($39,000)	Cost-effective	Academic resources	New Zealand	75
				Dominant					
**Pink** 2013 [40]	*CYP2C9* and *VKORC1*	No	QALY	£13,200 ($20,600)	£20,000–30,000 ($31,200–46,700)	Cost-effective	Academic resources	UK	93
**You** 2012 [41]	*CYP2C9* and *VKORC1*	No	QALY	*Dominated by genotype–guided approach*	$50,000	Dabigatran 150 mg seems to be cost-effective	No funding	USA	84
				*$13*,*800 per QALY*					
				*Dominated by dabigatran 150 mg*					
**You** 2014 [42]	*CYP2C9* and *VKORC1*	No	QALY	$2,800 per QALY	$50,000	Cost-effective	Research Grants Council of the Hong Kong special administrative Region, China	USA	70
**de Lima Lopes** 2012 [43]	*EGFR*	No	QALY	Dominant	n.a.	Dominant	AstraZeneca Pte Ltd.	Singapore	87
**Handorf** 2012 [44]	*EGFR*	No	QALY	$110,600	$100,000	Cost-effective	OSI Pharmaceuticals/Genentech	USA	90
**Zhu** 2013 [45]	*EGFR*	No	QALY and LYG	$57,000 per QALY	< 3 times the GDP per capita of China ($16,300)	Not cost-effective*	Shanghai Health Bureau	China	90
				$35,300 per LYG					
**Kauf** 2010 [46]	*HLA-B* *5701	No	HSR avoided	$300 (60 days’ time horizon)	no number.	Cost-effective	Glaxo Smith Kline, Inc.	USA	87
**Rattanavipa-pong** 2013 [47]	HLA-B*1502	Yes	QALY	***Epilepsy patients***	THB120,000 ($3,634)	PGx test is cost-effective for neuropathic pain but not for epilepsy	Academic resources	Thailand	70
				*THB220*,*000 ($6*,*700)*					
				*THB32*,*522*,*000 ($984*,*800)*					
				***Neuropathic pain patients***					
				*THB130*,*000 ($3*,*900)-*					
				*THB35*,*877*,*00 ($108*,*600)*					
**Liu** 2012 [48]	IL-28B	No	QALY	$50,400	No number	Not clear	Academic and governmental	USA	83
**Greeley** 2011 [49]	*KCNJ11* and *ABCC8*	Yes	QALY	Dominant	n.a.	Dominant	Academic resources	USA	56
**Parthan** 2013 [50]	*KIF6*	No	QALY	$45,000	$100,000	May be cost effective	Celera corporation	USA	83
**Vijayara-ghavan** 2012 [51]	*KRAS*	Yes	LYS	Cost saving	No number	Cost-saving in both US and Germany	Roche Molecular Systems, Inc.	USA and Germany	75
				Cost saving					
				$35,500 per LYS					
**Hagaman** 2010 [53]	*TPMT*	No	QALY	$29,700	$50,000	Cost-effective	Unknown	USA	64
**Thompson**. 2014 [52]	*TPMT*	No	QALY	Negative ICER	n.a.	Cost-saving but also health loss	Department of Health UK	UK	88
**Pichereau** 2010 [54]	UGT1A1 *28	No	neutropenia avoided	€900–1,100 ($1100–1300)	No number	Cost-effective	No specific financial support for this study	France	84

ABCC, ATP-binding cassette transporter sub family C; APOE, apolipoprotein-E; CYP, cytochrome P-450; EGFR, epidermal growth factor receptor; GPD: gross domestic product; HLA, human leukocyte antigen; HTTLPR, serotonin-transporter-linked polymorphic region; ICER, incremental cost-effectiveness ratio; KCNJ, Potassium inwardly-rectifying channel, subfamily J; KRAS, Kirsten rat sarcoma viral oncogene homolog; LYG, life-years-gained; LYS, life-years-saved; PGx, pharmacogenetic; QALW, quality-adjusted-life-week; QALY, quality-adjusted-life-year; THB, Thai Baht; TPMT, thiopurine S-methyltransferase; UGT, UDP-glucuronosyltransferase; UK, United Kingdom; VKORC, Vitamin K epoxide reductase complex

* With the gefitinib patient assistance program (sponsored therapy after first six months) it might be a cost-effective treatment option.

### Costs of PGx tests

When comparing the costs of the PGx tests, considerable differences between the costs of the tests were observed. This is not surprising, since technology of genetic testing is developing and costs are likely to be further reduced in the future. This was demonstrated by two studies of which one was performed in 2012 [41) and one in 2013 [40]. The costs for this particular screening test, US$72 and £20 respectively, were considerably lower compared to screening costs in studies performed in 2009 and 2010, which ranged from US$175 until US$575, respectively [63,78,96]. Another substantial difference was seen in the costs for the *CYP2C9* and *VKORC1* tests, which ranged from £20 to US$575 [40,78].

### Sensitivity analysis

Until 2008, only 5 out of the 31(16%) studies performed both a univariate and probabilistic sensitivity analysis, whereas since 2008 this were 35 out of the 49 (71%) studies (Fig 2B). One of the major uncertainty factors was the lack of robust clinical evidence for clinical utility of the PGx test itself. Hence, almost all authors had to define assumptions which were sometimes solely based on expert opinion. Other frequently mentioned uncertainty factors were the costs of the PGx tests and their real-world utility and performance. As a consequence of the uncertainty around the included genotyping costs, several studies included a costs range in their analysis. For example, Schackman *et al*. (2013) demonstrated that at a test cost of US$107, genetic testing was not cost-effective. However, at a price of US$10 per test, the PGx test was cost-effective [29]. Although variance in genotyping costs was frequently found to have a major impact on the ICER, 11 out of the 38 studies (30%) assessed in this update did not include a range of genotype costs in their sensitivity analysis (S1 Table). With respect to drug costs, some studies showed that drug driven costs did not influence the study’s outcome. For example, Crespin *et al*. (2011) showed in their sensitivity analysis that even if the costs of the drugs guided by PGx testing dropped substantially, the non-PGx-test-guided drug remained cost-effective [37].

### Genes investigated and analytical validity of PGx test

**Fig 3:**
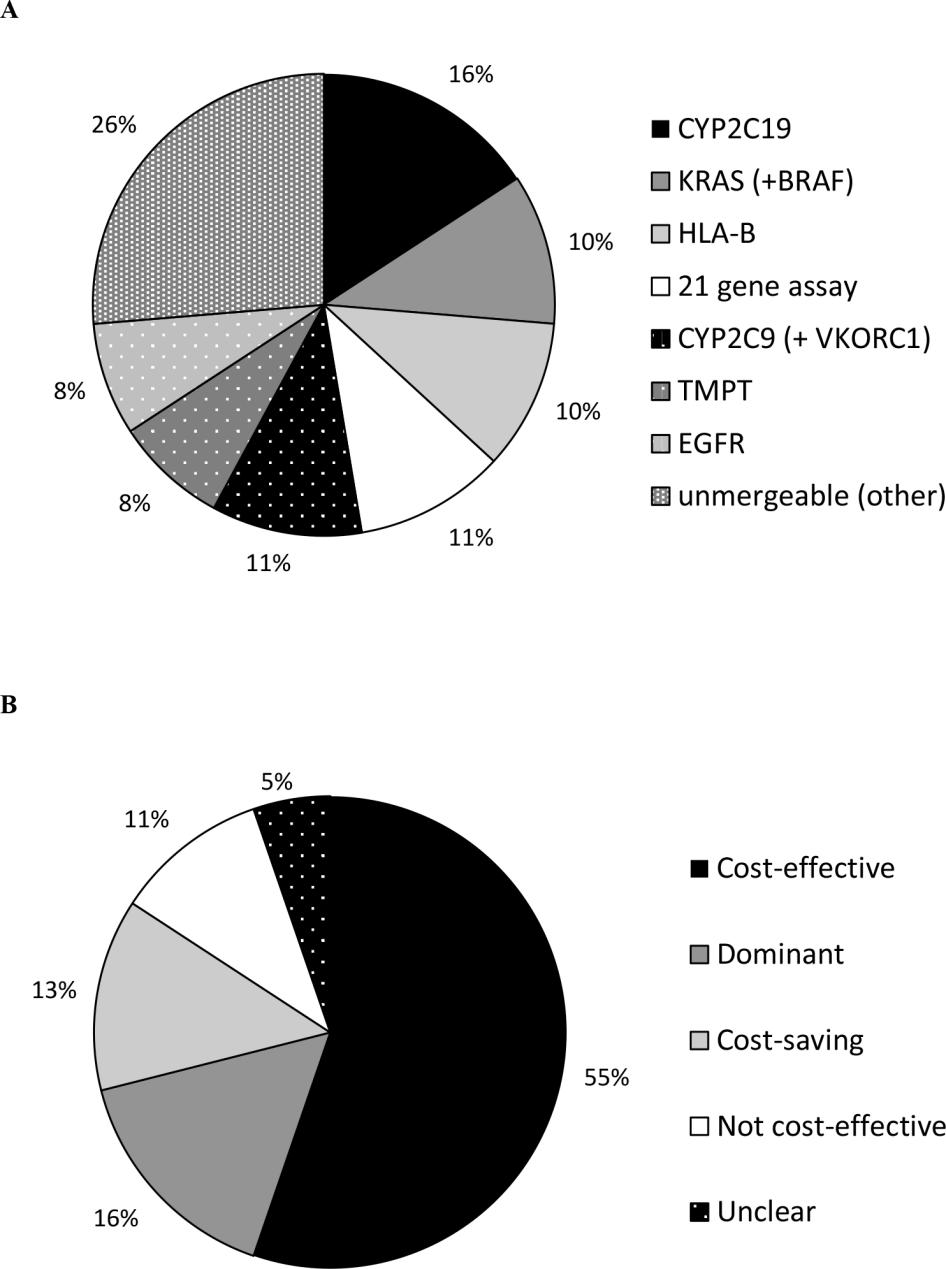
Genes analysed (A) and study outcomes (B) of papers published between August 2010 and September 2014.

In the period from 2000 until 2014, the majority of studies investigated the *TPMT* gene (17 studies). Among the studies presented in this update (i.e. from August 2010 onwards), most investigations concerned the *CYP2C19* screening tests (Fig 3A). We noticed that the *CYP2C19* gene was studied in many different scenarios. Some studies assessed if the use of a *CYP2C19* independent drug like prasugrel or ticagrelor would be a cost-effective treatment option [35,36,38]. Similar results were found for *CYP2C9* and *VKORC1* testing in combination with warfarin treatment, which was compared with *CYP2C9* and *VKORC1* independent novel oral anticoagulants, like dabigatran.[41].

In general the analytical validity of PGx tests is high (>95%), nevertheless variation in the analytical validity will in combination with the prevalence of a certain genetic trait determine the predictive value of a test [97]. This can be of influence on the cost-effectiveness and was nicely demonstrated by Kauf *et al*., who identified the negative predictive value as an important input parameter for the costs-effectiveness of HLA-B*5701 genotyping in their sensitivity analysis [46]. Nevertheless, underlying assumptions about the analytical validity of the test were not reported. It was found that only 11 out of the 38 (30%) studies included since 2010, did report underlying analytical validity of the PGx test (Table 1).

### Outcome of the studies

There were 21 out of 80 (26%) studies which concluded that PGx testing was dominant (i.e. resulting in clinical benefits as well as cost-savings). From 2010 onwards, most authors concluded that PGx testing was cost-effective, while only four studies concluded that it was not cost-effective (Fig 3B). In the period from 2010 until 2014, several studies provided the specific conditions at which genetic testing might become cost-effective (Tables 1and 2). For example, Dong *et al*. (2012) and Rattanavipapong *et al*. (2013) showed that the PGx tests could be cost-effective depending on either the patient population or the disease [23,47]. Due to an imperfect capability of PGx tests to differentiate between carriers of a genetic variant, some patients might be misclassified and receive suboptimal treatment. As a result, two studies assessing *KRAS* and *BRAF* testing and one study assessing *TMPT* testing found the PGx testing strategy was cost-saving, but with a small health loss compared to the non-testing strategy [25,26,52].

### Time horizon and discounting

To capture all costs, savings and effects of an intervention, a lifelong time horizon often seems the best time horizon, although in some scenarios it may be argued that a shorter period is acceptable. Before 2008, 12 out of 26 studies (46%) applied a time horizon of 12 months, and only five (19%) studies applied a lifelong time window. Among the studies published since 2010, there was a broad range in the applied time horizons, from two weeks until lifetime (S1 Table). In addition, out of the 38 studies 17 (45%) applied a lifelong time horizon, six (16%) studies applied a 30 year time horizon of which two studies also used a lifetime horizon. To deal with uncertainty around the appropriated time horizon, different time horizons can be used. We found that six out of 38 studies (16%) varied their time horizons [18,20,26,38,46,49]. In this way, insight into short- and long-term outcomes was given.

Discounting was applied for almost all studies published after 2006 that applied a time horizon longer than one year. The majority of the studies used discounting at 3% annually for costs and effects similarly (S1 Table).

### Discussion of limitations

The limitations and uncertainties of an economic analysis have to be acknowledged in order to judge the study on its merits. Nearly all of the newly included papers (i.e. published between August 2010 and October 2014) discussed their limitations, uncertainties and possible shortcomings of their economic analysis (S2 Table). The topic most commonly discussed was the lack of solid clinical evidence. As a result, many studies had to make assumptions or relied on experts’ opinions. However, as mentioned before, most authors did include different efficacy scenarios in their sensitivity analysis. Not all papers gave clear information about assumptions concerning sensitivity and specificity of the PGx tests, and only three out of 38 (8%) mentioned these assumptions as a limitation [45,49,51]. Another typical limitation for the effects of PGx tests is the time in which test results will become available for clinical decision making. In current analyses, test results were often assumed to be immediately available. However, this might be an unrealistic assumption and only one out of 38 studies explicitly mentioned this assumption as a limitation [23]. More general limitations were the lack of data with respect to heterogeneity in patient populations, hampering extrapolation of results to patients of different ethnicities, subpopulations and/or country specific populations. Moreover, several papers mentioned the difficulties in extrapolating long-term clinical utility results from the short-term clinical trials and lab studies.

### Role of funding

Out of the 78 selected studies, 11 (14%) were funded by pharmaceutical companies. Before 2008, none of the studies was (directly) funded by pharmaceutical companies. In 2008 and 2009 only two (12.5%) out of 16 studies published in that period were funded by a pharmaceutical company [70,71]. Between August 2010 and September 2014, 9 (24%) out of the 38 selected studies were funded by pharmaceutical companies (Tables 1 and 2). Regarding outcomes and conclusions all of these studies concluded that PGx tests were dominant, cost-saving, or cost-effective. Among the remaining studies which were funded by other resources, 14% concluded PGx tests were not cost-effective.

### Quality assessment

The studies included through this update received a quality score according to the QHES. There was 2% disagreement between the reviewers for which consensus was reached through discussion. The average quality score was 76. The score which was given to each study is shown in Tables 1 and 2. The majority of the studies (71%) were of high quality. On average, studies concerning testing for *EGFR* received the highest rating and studies about the 21-gene assay received the lowest rating. Some items were scored negative for the majority of the studies. One of them was the item concerning the perspective of the analysis (i.e. societal, health care payers, etcetera). Most studies did not explain why the perspective of the analysis was chosen. In addition, low scoring was received for separate reporting of the short-and long-term outcomes. Most studies did not make such a distinction. Lastly, the direction and magnitude of potential biases were often not discussed.

## Discussion

### Principal findings

Since 2004, there is an increase in the number of studies evaluating the economic value of PGx tests and this increase accelerated from 2008 onwards. There were not that many economic evaluations of PGx tests available as one might expect given the unravelling of the whole human genome in 2003, the clinical possibilities, and the fast development and decreasing costs of genetic tests [98]. This could be related to limited implementation of pharmacogenetic knowledge into daily clinical practice [5]. Reasons for this are the uncertainty about clinical relevance, concerns about the availability of genetic data and considerable differences in cost-effectiveness which are found, in particular between countries [5,99,100].

Many studies included through this update did not assess the intrinsic value of the PGx test itself, but compared a PGx test treatment combination with an alternative treatment. For example, the tests for *CYP2C19* or, both *CYP2C9* and *VKORC1* were incorporated in several models as the current treatment option in combination with clopidogrel or warfarin treatment, respectively. The alternative treatment options, which were assessed in the included studies, were independent of pharmacogenetics and were found to be cost-effective. Therefore, in cost-effectiveness analysis of antithrombotic therapy, there seems an ongoing movement away from pharmacogenetic testing, towards treatment options with compounds that are, so far, considered to be independent of pharmacogenetics.

Before 2008, most analyses were cost-effectiveness analyses, however since 2008 there has been a trend towards the use of cost-utility analyses. Cost-utility analysis is currently considered as the preferred type of analysis for health care choices as is advised in several national guidelines, although other types can be suitable depending on the specific study ([1,102]. Before 2008, most studies performed only a simple univariate sensitivity analysis, if any [9]. We found that more recent studies performed both univariate *and* probabilistic sensitivity analyses. This combination is also advised in several national guidelines for pharmacoeconomic evaluation and is a part of the QHES checklist [16,101,102]. As a result of these more comprehensive sensitivity analyses, the quality of economic evaluations appears to be improved over the last decade. However, we found that differences in genotyping costs were not always included in the sensitivity analysis which leaves room for improvement.

Among the new studies included in this update (i.e. since 2010), considerable differences in the length of the time horizon were noticed, varying from two weeks to lifelong. A time horizon shorter than a year was primarily related to the expected relevant clinical outcomes. Interestingly, there were differences in the time horizon between studies investigating the same genes. For example, Kazi *et al*. and Panattoni *et al*. applied a lifelong time horizon [34,39], whereas others applied a time horizon of 15 months [35,38]. All studies discussed at least some limitations and a lack of robust clinical evidence was most frequently mentioned as an important limitation contributing to uncertainty in the analysis. Although studies frequently made assumptions about analytical validity of PGx tests as well as about the rapid clinical availability, these were often not discussed as limitations or studied in sensitivity or scenario analyses. Besides the analytical validity, the clinical validity of PGx tests is important. In general this is the same as an effect estimate of a PGx test. As such, the clinical validity is correctly embedded in an economic evaluation. However, this does not apply for all PGx tests, because some PGx tests involve an analysis of multiple genetic variations. These variations can all contribute to a similar genotype prediction and therefore the clinical validity depends on the number and type of variant alleles analysed. For example, *CYP2C19* genotyping which depends on general molecular genetic analysis of single nuclear polymorphisms to detect variant alleles. Several studies included in this review studied only the CYP2C19*2 allele [37–39]. However, other allele like the *3 allele can also effect *CYP2C19* activity and give a similar clinical effect as the *2 allele [103]. When more variant alleles are analysed the clinical validity of the PGx test will increase, although some variants are rare and will contribute little. With the ongoing and rapid increase of knowledge about variant alleles, it is important economic evaluations report the variant alleles on which their assumptions about effects of genotyping (i.e. clinical validity) were based. For the example of *CYP2C19*, two out of the six studies involving the *CYP2C19* gene, did not specify which alleles were included [34,36]. This hampers the extrapolation of their findings towards populations of a different ethnicity and other laboratories.

Compared to studies published before 2008, studies were more commonly funded by pharmaceutical companies. Interestingly, all of these sponsored studies concluded that PGx tests were dominant, cost-saving, or cost-effective whereas the few studies which concluded otherwise were financed by academic, governmental, or unknown resources. It is known that studies funded by pharmaceutical companies publish more positive results when compared to studies funded by other resources which is in line with our results [104]. These positive biased results are not related towards the quality of the studies, but to the comparison which is made or publication bias [105]. For many of the economic evaluations in this review, assumptions about the effect of the genetic test were made. Furthermore, analytical validity was often not included in the model. This leaves room to bias result in favour of a preferred treatment strategy. Therefore, attention should be given toward assumptions about these aspects, especially when studies are funded by pharmaceutical companies or if the funding is not reported.

Most studies included in this review concluded that the application of PGx tests was cost-effective. Yet, the conclusions were not unambiguous, often due to the uncertainties in the economic models. Another reason for this was that most of the newly included studies did not assess the intrinsic value of the PGx test itself, but a scenario involving one or more PGx tests. Although such estimates are important for the economic impact of the application of personalized medicine, they do not provide information about the cost-effectiveness of the PGx test itself and therefore outcomes can be different. To improve generalizability between studies, an additional scenario analysis in which equal costs and efficacy of the compared treatment strategies are assumed, to assess the intrinsic value of the PGx tests, could be used. Such an approach would especially be interesting when the comparator drug is under patent and drug costs are likely to decrease in the future. Another aspect creating different conclusions was the genetic variety between study populations. This was clearly described by some papers which mentioned the specific conditions like a specific geographic region or a disease for which the genetic testing was cost-effective. For example, PGx testing for HLA-B*5702 was cost-effective in Singaporean Chinese and Malays, but not in Singaporean Indians [23]. In addition, Rattanavipapong *et al*. (2013) found that HLA-B*5702 testing was cost-effective in epileptic patients, but not in neuropathic pain patients [47].

### Comparison with previous literature reviews

For the new studies included in this update (i.e. since 2010), an average QHES-quality score of 76 was found. This was is in line with the results from the review from Wong *et al*. about pharmacoeconomics of PGx tests. They found an average quality score of 77 [13]. However, it was lower compared to a related review of Djalalov *et al*., who found an average quality score of 90 [10]. This is likely due to the subjective nature of some items in the quality assessment. For example, ‘item 3’ asks if the used estimates for the analysis were from the best available source, and is therefore quite sensitive to the interpretation of the reviewer [16]. Previous review studies pointed out that the methodology of economic evaluations of PGx tests is often heterogeneous and of insufficient quality [9,11]. Assasi *et al*. found that economic PGx studies which were of low quality (i.e. QHES score of < 50), frequently failed to handle uncertainty, did not inform about the study’s limitations, and did not discuss direction and magnitude of potential uncertainty [11]. Among the studies included in this update (i.e. since 2010), most of the authors discussed uncertainties and except for the study by Tiamkao *et al*., all incorporated uncertainty in a sensitivity analysis [24]. However, direction and magnitude of potential bias was still not sufficiently discussed and remains a major point for improvement.

### Limitations of our approach

This review has some limitations. Firstly, we did not include studies from other databases than PubMed or grey literature and we only included English written studies. Therefore some studies might have been missed. In general, studies that are not indexed in MEDLINE or written in English do not have a large impact on reviews’ outcomes [106]. Nevertheless, these studies are frequently of lower quality, and therefore the average quality of the studies included in our review might have been slightly overestimated. However, based on the comparison with other reviews we found a slightly lower average quality score. Lastly, publication bias can always influence the findings of a review. Therefore cost-effectiveness of PGx tests could be overestimated.

### Recommendations

Based on the quality assessment, reporting of the reasons behind the chosen perspective and the type of economic model can improve the quality of economic evaluations of PGx tests. In addition, reporting of both short-and long-term outcomes and the influence of potential bias, in terms of direction and magnitude on the cost-effectiveness estimates could be improved. Although a substantial and persistent increase in the use of both univariate and probabilistic sensitivity analyses was observed since 2008, there is still room for improvement by using a combination of these techniques instead of one technique, among several studies published since 2008. Publication bias or biased comparators might favour cost-effectiveness of PGx tests. Among studies funded by companies with conflicting interests, the risk on this kind of bias should be critically assessed. In our previous reviews the main limitation that was identified was the unavailability of clinical evidence [1,9]. Although this remains an important issue, based on our new findings we can add some recommendations which are more applicable to implementation of PGx tests in clinical practice. First, the clinical validity of a test, i.e. the capability of the test to predict phenotypes with a clinical effect and the analytical validity should be reported as is recommended by the US Academy of Managed Care Pharmacy [107]. Both parameters should be included in a sensitivity analysis. In addition, the variant alleles on which these parameters were based should be reported. For some PGx tests, these estimates might be unknown. In this case, a better approach towards this problem would be inclusion of an univariate sensitivity analysis with different cut-off values for the analytical and clinical validity of the PGx test. In this way, a minimum for the analytical and clinical validity can be generated. Note that with an increase in analysed alleles and as such the clinical validity, the price for the PGx test usually increases as well. Secondly, different turnaround times in which PGx test results would become available for healthcare professionals after requesting the test would be very informative. If direct availability of the genetic test is assumed, for example in the case of pre-emptive genotyping, this should be clearly stated in the method section. This way, the generalizability of results to other countries where PGx tests are available would improve. In addition, a range of costs for the genetic test should be evaluated in univariate and if applicable probabilistic sensitivity analyses. Furthermore, we recommend the addition of a scenario analysis in which drug costs between comparator groups are equalized to give information about the intrinsic value of the PGx test itself.

## Conclusion

Application of PGx tests was mostly found to be a cost-effective or cost-saving strategy, although some studies concluded otherwise which underlines the importance of future studies assessing the cost-effectiveness of PGx tests. We found that only the minority of recent pharmacoeconomic evaluations assessed the intrinsic value of the PGx tests. New compounds that are not affected by genetics, are emerging as cost-effective alternatives for pharmacogenetic testing strategies. Over the last decade, there was an increase in the number of studies and in the reporting of quality associated characteristics. Due to rapid development in analytical techniques, reporting of analytical and clinical validity of the assessed PGx test is recommended for future evaluations. Furthermore robust clinical evidence regarding PGx tests’ efficacy is warranted.

## Supporting Information

S1 PRISMA Checklist (DOCX)

S1 Table Overview of pharmacoeconomic PGx studies published between August 2010 and September 2014 analysing the intrinsic value of a PGx test (A), or comparing different treatment strategies involving PGx testing (B). (DOCX)

S2 Table Main limitations that were discussed in the papers published between August 2010 and September 2014. (DOCX)

## References

[1] Vegter S, Jansen E, Postma MJ, Boersma C. Economic evaluations of pharmacogenetic and genomic screening programs: update of the literature. Drug Development Research 2010;71:492–501.

[2] Cascorbi I, Bruhn O, Werk AN. Challenges in pharmacogenetics. Eur J Clin Pharmacol 2013 5;69 Suppl 1:17–23. 10.1007/s00228-013-1492-x 23640184

[3] Ross S, Anand SS, Joseph P, Pare G. Promises and challenges of pharmacogenetics: an overview of study design, methodological and statistical issues. JRSM Cardiovasc Dis 2012 4 5;1(1): 10.1258/cvd.2012.012001 PMC3738322 24175062

[4] Garrison LP Jr, Carlson RJ, Carlson JJ, Kuszler PC, Meckley LM, Veenstra DL. A review of public policy issues in promoting the development and commercialization of pharmacogenomic applications: challenges and implications. Drug Metab Rev 2008;40(2):377–401. 10.1080/03602530801952500 18464050

[5] Johnson JA, Burkley BM, Langaee TY, Clare-Salzler MJ, Klein TE, Altman RB. Implementing personalized medicine: development of a cost-effective customized pharmacogenetics genotyping array. Clin Pharmacol Ther 2012 10;92(4):437–439. 10.1038/clpt.2012.125 22910441 PMC3454443

[6] Phillips KA, Ann Sakowski J, Trosman J, Douglas MP, Liang SY, Neumann P. The economic value of personalized medicine tests: what we know and what we need to know. Genet Med 2014 3;16(3):251–257. 10.1038/gim.2013.122 24232413 PMC3949119

[7] Husereau D, Marshall DA, Levy AR, Peacock S, Hoch JS. Health technology assessment and personalized medicine: are economic evaluation guidelines sufficient to support decision making? Int J Technol Assess Health Care 2014 4;30(2):179–187. 10.1017/S0266462314000142 24806420

[8] Hatz MH, Schremser K, Rogowski WH. Is individualized medicine more cost-effective? A systematic review. Pharmacoeconomics 2014 5;32(5):443–455. 10.1007/s40273-014-0143-0 24574059

[9] Vegter S, Boersma C, Rozenbaum M, Wilffert B, Navis G, Postma MJ. Pharmacoeconomic evaluations of pharmacogenetic and genomic screening programmes: a systematic review on content and adherence to guidelines. Pharmacoeconomics 2008;26(7):569–587. 18563949 10.2165/00019053-200826070-00005

[10] Djalalov S, Musa Z, Mendelson M, Siminovitch K, Hoch J. A review of economic evaluations of genetic testing services and interventions (2004–2009). Genet Med 2011 2;13(2):89–94. 10.1097/GIM.0b013e3182003294 21273949

[11] Assasi N, Schwartz L, Tarride JE, Goeree R, Xie F. Economic evaluations conducted for assessment of genetic testing technologies: a systematic review. Genet Test Mol Biomarkers 2012 11;16(11):1322–1335. 10.1089/gtmb.2012.0178 23009569

[12] Beaulieu M, de Denus S, Lachaine J. Systematic review of pharmacoeconomic studies of pharmacogenomic tests. Pharmacogenomics 2010 11;11(11):1573–1590. 10.2217/pgs.10.145 21121811

[13] Wong WB, Carlson JJ, Thariani R, Veenstra DL. Cost effectiveness of pharmacogenomics: a critical and systematic review. Pharmacoeconomics 2010;28(11):1001–1013. 10.2165/11537410-000000000-00000 20936884

[14] Phillips KA, Ann Sakowski J, Trosman J, Douglas MP, Liang SY, Neumann P. The economic value of personalized medicine tests: what we know and what we need to know. Genet Med 2014 3;16(3):251–257. 10.1038/gim.2013.122 24232413 PMC3949119

[15] Shiroiwa T, Motoo Y, Tsutani K. Cost-effectiveness analysis of KRAS testing and cetuximab as last-line therapy for colorectal cancer. Mol Diagn Ther 2010 12 1;14(6):375–384. 10.2165/11587610-000000000-00000 21275455

[16] Chiou CF, Hay JW, Wallace JF, Bloom BS, Neumann PJ, Sullivan SD, et al Development and validation of a grading system for the quality of cost-effectiveness studies. Med Care 2003 1;41(1):32–44. 12544542 10.1097/00005650-200301000-00007

[17] Moher D, Liberati A, Tetzlaff J, Altman DG, PRISMA Group. Preferred reporting items for systematic reviews and meta-analyses: the PRISMA statement. PLoS Med 2009 7 21;6(7):e1000097 10.1371/journal.pmed.1000097 19621072 PMC2707599

[18] Klang SH, Hammerman A, Liebermann N, Efrat N, Doberne J, Hornberger J. Economic implications of 21-gene breast cancer risk assay from the perspective of an Israeli-managed health-care organization. Value Health 2010 Jun-Jul;13(4):381–387. 10.1111/j.1524-4733.2010.00724.x 20412544

[19] Bacchi CE, Prisco F, Carvalho FM, Ojopi EB, Saad ED. Potential economic impact of the 21-gene expression assay on the treatment of breast cancer in Brazil. Rev Assoc Med Bras 2010 Mar-Apr;56(2):186–191. 20498993 10.1590/s0104-42302010000200017

[20] Hall PS, McCabe C, Stein RC, Cameron D. Economic evaluation of genomic test-directed chemotherapy for early-stage lymph node-positive breast cancer. J Natl Cancer Inst 2012 1 4;104(1):56–66. 10.1093/jnci/djr484 22138097

[21] Vanderlaan BF, Broder MS, Chang EY, Oratz R, Bentley TG. Cost-effectiveness of 21-gene assay in node-positive, early-stage breast cancer. Am J Manag Care 2011;17(7):455–464. 21819166

[22] Verhoef TI, Redekop WK, Veenstra DL, Thariani R, Beltman PA, van Schie RM, et al Cost-effectiveness of pharmacogenetic-guided dosing of phenprocoumon in atrial fibrillation. Pharmacogenomics 2013 6;14(8):869–883. 10.2217/pgs.13.74 23746182

[23] Dong D, Sung C, Finkelstein EA. Cost-effectiveness of HLA-B*1502 genotyping in adult patients with newly diagnosed epilepsy in Singapore. Neurology 2012 9 18;79(12):1259–1267. 10.1212/WNL.0b013e31826aac73 22955130

[24] Tiamkao S, Jitpimolmard J, Sawanyawisuth K, Jitpimolmard S. Cost minimization of HLA-B*1502 screening before prescribing carbamazepine in Thailand. Int J Clin Pharm 2013 8;35(4):608–612. 10.1007/s11096-013-9777-9 23649893

[25] Blank PR, Moch H, Szucs TD, Schwenkglenks M. KRAS and BRAF mutation analysis in metastatic colorectal cancer: a cost-effectiveness analysis from a Swiss perspective. Clin Cancer Res 2011 10 1;17(19):6338–6346. 10.1158/1078-0432.CCR-10-2267 21807639

[26] Behl AS, Goddard KA, Flottemesch TJ, Veenstra D, Meenan RT, Lin JS, et al Cost-effectiveness analysis of screening for KRAS and BRAF mutations in metastatic colorectal cancer. J Natl Cancer Inst 2012 12 5;104(23):1785–1795. 10.1093/jnci/djs433 23197490 PMC3514165

[27] Shiffman D, Slawsky K, Fusfeld L, Devlin JJ, Goss TF. Cost-effectiveness model of use of genetic testing as an aid in assessing the likely benefit of aspirin therapy for primary prevention of cardiovascular disease. Clin Ther 2012 6;34(6):1387–1394. 10.1016/j.clinthera.2012.04.004 22560621

[28] Donnan JR, Ungar WJ, Mathews M, Hancock-Howard RL, Rahman P. A cost effectiveness analysis of thiopurine methyltransferase testing for guiding 6-mercaptopurine dosing in children with acute lymphoblastic leukemia. Pediatr Blood Cancer 2011 8;57(2):231–239. 10.1002/pbc.22936 21344614

[29] Schackman BR, Haas DW, Becker JE, Berkowitz BK, Sax PE, Daar ES, et al Cost-effectiveness analysis of UGT1A1 genetic testing to inform antiretroviral prescribing in HIV disease. Antivir Ther 2013;18(3):399–408. 10.3851/IMP2500 23264445 PMC3744167

[30] Olgiati P, Bajo E, Bigelli M, De Ronchi D, Serretti A. Should pharmacogenetics be incorporated in major depression treatment? Economic evaluation in high- and middle-income European countries. Prog Neuropsychopharmacol Biol Psychiatry 2012 1 10;36(1):147–154. 10.1016/j.pnpbp.2011.08.013 21911028

[31] Serretti A, Olgiati P, Bajo E, Bigelli M, De Ronchi D. A model to incorporate genetic testing (5-HTTLPR) in pharmacological treatment of major depressive disorders. World J Biol Psychiatry 2011 10;12(7):501–515. 10.3109/15622975.2011.572998 21595526

[32] Reed SD, Scales CD Jr, Stewart SB, Sun J, Moul JW, Schulman KA, et al Effects of family history and genetic polymorphism on the cost-effectiveness of chemoprevention with finasteride for prostate cancer. J Urol 2011 3;185(3):841–847. 10.1016/j.juro.2010.10.078 21239023 PMC3059593

[33] Djalalov S, Yong J, Beca J, Black S, Saposnik G, Musa Z, et al Genetic testing in combination with preventive donepezil treatment for patients with amnestic mild cognitive impairment: an exploratory economic evaluation of personalized medicine. Mol Diagn Ther 2012 12;16(6):389–399. 10.1007/s40291-012-0010-7 23188525

[34] Kazi DS, Garber AM, Shah RU, Dudley RA, Mell MW, Rhee C, et al Cost-effectiveness of genotype-guided and dual antiplatelet therapies in acute coronary syndrome. Ann Intern Med 2014 2 18;160(4):221–232. 24727840 10.7326/M13-1999

[35] Reese ES, Daniel Mullins C, Beitelshees AL, Onukwugha E. Cost-effectiveness of cytochrome P450 2C19 genotype screening for selection of antiplatelet therapy with clopidogrel or prasugrel. Pharmacotherapy 2012 4;32(4):323–332. 10.1002/j.1875-9114.2012.01048 22461122 PMC3883873

[36] Sorich MJ, Horowitz JD, Sorich W, Wiese MD, Pekarsky B, Karnon JD. Cost-effectiveness of using CYP2C19 genotype to guide selection of clopidogrel or ticagrelor in Australia. Pharmacogenomics 2013 12;14(16):2013–2021. 10.2217/pgs.13.164 24279856

[37] Crespin DJ, Federspiel JJ, Biddle AK, Jonas DE, Rossi JS. Ticagrelor versus genotype-driven antiplatelet therapy for secondary prevention after acute coronary syndrome: a cost-effectiveness analysis. Value Health 2011 6;14(4):483–491. 10.1016/j.jval.2010.11.012 21669373 PMC3384486

[38] Lala A, Berger JS, Sharma G, Hochman JS, Scott Braithwaite R, Ladapo JA. Genetic testing in patients with acute coronary syndrome undergoing percutaneous coronary intervention: a cost-effectiveness analysis. J Thromb Haemost 2013 1;11(1):81–91. 10.1111/jth.12059 23137413

[39] Panattoni L, Brown PM, Te Ao B, Webster M, Gladding P. The cost effectiveness of genetic testing for CYP2C19 variants to guide thienopyridine treatment in patients with acute coronary syndromes: a New Zealand evaluation. Pharmacoeconomics 2012 11 1;30(11):1067–1084. 10.2165/11595080-000000000-00000 22974536

[40] Pink J, Pirmohamed M, Lane S, Hughes DA. Cost-effectiveness of pharmacogenetics-guided warfarin therapy vs. alternative anticoagulation in atrial fibrillation. Clin Pharmacol Ther 2014 2;95(2):199–207. 10.1038/clpt.2013.190 24067746

[41] You JH, Tsui KK, Wong RS, Cheng G. Cost-effectiveness of dabigatran versus genotype-guided management of warfarin therapy for stroke prevention in patients with atrial fibrillation. PLoS One 2012;7(6):e39640 10.1371/journal.pone.0039640 22745801 PMC3382133

[42] You JH. Pharmacogenetic-guided selection of warfarin versus novel oral anticoagulants for stroke prevention in patients with atrial fibrillation: a cost-effectiveness analysis. Pharmacogenetics and genomics 2014;24(1):6–14. 10.1097/FPC.0000000000000014 24168919

[43] de Lima Lopes G Jr, Segel JE, Tan DS, Do YK, Mok T, Finkelstein EA. Cost-effectiveness of epidermal growth factor receptor mutation testing and first-line treatment with gefitinib for patients with advanced adenocarcinoma of the lung. Cancer 2012 2 15;118(4):1032–1039. 10.1002/cncr.26372 21792863

[44] Handorf EA, McElligott S, Vachani A, Langer CJ, Bristol Demeter M, Armstrong K, et al Cost effectiveness of personalized therapy for first-line treatment of stage IV and recurrent incurable adenocarcinoma of the lung. J Oncol Pract 2012 9;8(5):267–274. 10.1200/JOP.2011.000502 23277762 PMC3439225

[45] Zhu J, Li T, Wang X, Ye M, Cai J, Xu Y, et al Gene-guided gefitinib switch maintenance therapy for patients with advanced EGFR mutation-positive non-small cell lung cancer: an economic analysis. BMC Cancer 2013 1 29;13:39-2407-13-39. 10.1186/1471-2407-13-39 PMC3568065 23360224

[46] Kauf TL, Farkouh RA, Earnshaw SR, Watson ME, Maroudas P, Chambers MG. Economic efficiency of genetic screening to inform the use of abacavir sulfate in the treatment of HIV. Pharmacoeconomics 2010;28(11):1025–1039. 10.2165/11535540-000000000-00000 20575592

[47] Rattanavipapong W, Koopitakkajorn T, Praditsitthikorn N, Mahasirimongkol S, Teerawattananon Y. Economic evaluation of HLA-B*15:02 screening for carbamazepine-induced severe adverse drug reactions in Thailand. Epilepsia 2013 9;54(9):1628–1638. 10.1111/epi.12325 23895569

[48] Liu S, Cipriano LE, Holodniy M, Owens DK, Goldhaber-Fiebert JD. New protease inhibitors for the treatment of chronic hepatitis C: a cost-effectiveness analysis. Ann Intern Med 2012 2 21;156(4):279–290. 10.7326/0003-4819-156-4-201202210-00005 22351713 PMC3586733

[49] Greeley SA, John PM, Winn AN, Ornelas J, Lipton RB, Philipson LH, et al The cost-effectiveness of personalized genetic medicine: the case of genetic testing in neonatal diabetes. Diabetes Care 2011 3;34(3):622–627. 10.2337/dc10-1616 21273495 PMC3041194

[50] Parthan A, Leahy KJ, O'Sullivan AK, Iakoubova OA, Bare LA, Devlin JJ, et al Cost effectiveness of targeted high-dose atorvastatin therapy following genotype testing in patients with acute coronary syndrome. Pharmacoeconomics 2013 6;31(6):519–531. 10.1007/s40273-013-0054-5 23585310

[51] Vijayaraghavan A, Efrusy MB, Goke B, Kirchner T, Santas CC, Goldberg RM. Cost-effectiveness of KRAS testing in metastatic colorectal cancer patients in the United States and Germany. Int J Cancer 2012 7 15;131(2):438–445. 10.1002/ijc.26400 21898389

[52] Thompson AJ, Newman WG, Elliott RA, Roberts SA, Tricker K, Payne K. The cost-effectiveness of a pharmacogenetic test: a trial-based evaluation of TPMT genotyping for azathioprine. Value Health 2014 Jan-Feb;17(1):22–33. 10.1016/j.jval.2013.10.007 24438714

[53] Hagaman JT, Kinder BW, Eckman MH. Thiopurine S- methyltransferase [corrected] testing in idiopathic pulmonary fibrosis: a pharmacogenetic cost-effectiveness analysis. Lung 2010 4;188(2):125–132. 10.1007/s00408-009-9217-8 20066544

[54] Pichereau S, Le Louarn A, Lecomte T, Blasco H, Le Guellec C, Bourgoin H. Cost-effectiveness of UGT1A1*28 genotyping in preventing severe neutropenia following FOLFIRI therapy in colorectal cancer. J Pharm Pharm Sci 2010;13(4):615–625. 21486535 10.18433/j3wk5s

[55] Blank PR, Schwenkglenks M, Moch H, Szucs TD. Human epidermal growth factor receptor 2 expression in early breast cancer patients: a Swiss cost-effectiveness analysis of different predictive assay strategies. Breast Cancer Res Treat 2010 11;124(2):497–507. 10.1007/s10549-010-0862-7 20364309

[56] Carlson JJ, Garrison LP, Ramsey SD, Veenstra DL. The potential clinical and economic outcomes of pharmacogenomic approaches to EGFR-tyrosine kinase inhibitor therapy in non-small-cell lung cancer. Value Health 2009 Jan-Feb;12(1):20–27. 10.1111/j.1524-4733.2008.00415.x 18647257

[57] Chou WH, Yan FX, de Leon J, Barnhill J, Rogers T, Cronin M, et al Extension of a pilot study: impact from the cytochrome P450 2D6 polymorphism on outcome and costs associated with severe mental illness. J Clin Psychopharmacol 2000 4;20(2):246–251. 10770465 10.1097/00004714-200004000-00019

[58] Cosler LE, Lyman GH. Economic analysis of gene expression profile data to guide adjuvant treatment in women with early-stage breast cancer. Cancer Invest 2009 12;27(10):953–959. 10.3109/07357900903275217 19909009

[59] Costa-Scharplatz M, van Asselt AD, Bachmann LM, Kessels AG, Severens JL. Cost-effectiveness of pharmacogenetic testing to predict treatment response to angiotensin-converting enzyme inhibitor. Pharmacogenet Genomics 2007 5;17(5):359–368. 17429318 10.1097/01.fpc.0000236336.34175.e8

[60] Dendukuri N, Khetani K, McIsaac M, Brophy J. Testing for HER2-positive breast cancer: a systematic review and cost-effectiveness analysis. CMAJ 2007 5 8;176(10):1429–1434. 17485695 10.1503/cmaj.061011 PMC1863543

[61] Desta Z, Zhao X, Shin JG, Flockhart DA. Clinical significance of the cytochrome P450 2C19 genetic polymorphism. Clin Pharmacokinet 2002;41(12):913–958. 12222994 10.2165/00003088-200241120-00002

[62] Dubinsky MC, Reyes E, Ofman J, Chiou CF, Wade S, Sandborn WJ. A cost-effectiveness analysis of alternative disease management strategies in patients with Crohn's disease treated with azathioprine or 6-mercaptopurine. Am J Gastroenterol 2005 10;100(10):2239–2247. 16181376 10.1111/j.1572-0241.2005.41900.x

[63] Eckman MH, Rosand J, Greenberg SM, Gage BF. Cost-effectiveness of using pharmacogenetic information in warfarin dosing for patients with nonvalvular atrial fibrillation. Ann Intern Med 2009 1 20;150(2):73–83. 19153410 10.7326/0003-4819-150-2-200901200-00005

[64] Elkin EB, Weinstein MC, Winer EP, Kuntz KM, Schnitt SJ, Weeks JC. HER-2 testing and trastuzumab therapy for metastatic breast cancer: a cost-effectiveness analysis. J Clin Oncol 2004 3 1;22(5):854–863. 14990641 10.1200/JCO.2004.04.158

[65] Furuta T, Shirai N, Kodaira M, Sugimoto M, Nogaki A, Kuriyama S, et al Pharmacogenomics-based tailored versus standard therapeutic regimen for eradication of H. pylori. Clin Pharmacol Ther 2007 4;81(4):521–528. 17215846 10.1038/sj.clpt.6100043

[66] Gold HT, Hall MJ, Blinder V, Schackman BR. Cost effectiveness of pharmacogenetic testing for uridine diphosphate glucuronosyltransferase 1A1 before irinotecan administration for metastatic colorectal cancer. Cancer 2009 9 1;115(17):3858–3867. 10.1002/cncr.24428 19517472 PMC2853177

[67] Hughes DA, Vilar FJ, Ward CC, Alfirevic A, Park BK, Pirmohamed M. Cost-effectiveness analysis of HLA B*5701 genotyping in preventing abacavir hypersensitivity. Pharmacogenetics 2004 6;14(6):335–342. 15247625 10.1097/00008571-200406000-00002

[68] Kim SK, Jun JB, El-Sohemy A, Bae SC. Cost-effectiveness analysis of MTHFR polymorphism screening by polymerase chain reaction in Korean patients with rheumatoid arthritis receiving methotrexate. J Rheumatol 2006 7;33(7):1266–1274. 16758511

[69] Lehmann DF, Medicis JJ, Franklin PD. Polymorphisms and the pocketbook: the cost-effectiveness of cytochrome P450 2C19 genotyping in the eradication of Helicobacter pylori infection associated with duodenal ulcer. J Clin Pharmacol 2003 12;43(12):1316–1323. 14615467 10.1177/0091270003259389

[70] Lidgren M, Jonsson B, Rehnberg C, Willking N, Bergh J. Cost-effectiveness of HER2 testing and 1-year adjuvant trastuzumab therapy for early breast cancer. Ann Oncol 2008 3;19(3):487–495. 18065409 10.1093/annonc/mdm488

[71] Lidgren M, Wilking N, Jonsson B, Rehnberg C. Cost-effectiveness of HER2 testing and trastuzumab therapy for metastatic breast cancer. Acta Oncol 2008;47(6):1018–1028. 10.1080/02841860801901618 18607881

[72] Maitland-van der Zee AH, Klungel OH, Stricker BH, Veenstra DL, Kastelein JJ, Hofman A, et al Pharmacoeconomic evaluation of testing for angiotensin-converting enzyme genotype before starting beta-hydroxy-beta-methylglutaryl coenzyme A reductase inhibitor therapy in men. Pharmacogenetics 2004 1;14(1):53–60. 15128051 10.1097/00008571-200401000-00006

[73] Marra CA, Esdaile JM, Anis AH. Practical pharmacogenetics: the cost effectiveness of screening for thiopurine s-methyltransferase polymorphisms in patients with rheumatological conditions treated with azathioprine. J Rheumatol 2002 12;29(12):2507–2512. 12465143

[74] Meckley LM, Veenstra DL. Screening for the alpha-adducin Gly460Trp variant in hypertensive patients: a cost-effectiveness analysis. Pharmacogenet Genomics 2006 2;16(2):139–147. 16424826 10.1097/01.fpc.0000189801.96220.82

[75] Meckley LM, Gudgeon JM, Anderson JL, Williams MS, Veenstra DL. A policy model to evaluate the benefits, risks and costs of warfarin pharmacogenomic testing. Pharmacoeconomics 2010;28(1):61–74. 10.2165/11318240-000000000-00000 20014877

[76] Morelle M, Hasle E, Treilleux I, Michot JP, Bachelot T, Penault-Llorca F, et al Cost-effectiveness analysis of strategies for HER2 testing of breast cancer patients in France. Int J Technol Assess Health Care 2006 Summer;22(3):396–401. 16984069 10.1017/s0266462306051300

[77] Oh KT, Anis AH, Bae SC. Pharmacoeconomic analysis of thiopurine methyltransferase polymorphism screening by polymerase chain reaction for treatment with azathioprine in Korea. Rheumatology (Oxford) 2004 2;43(2):156–163. 12923290 10.1093/rheumatology/keh001

[78] Patrick AR, Avorn J, Choudhry NK. Cost-effectiveness of genotype-guided warfarin dosing for patients with atrial fibrillation. Circ Cardiovasc Qual Outcomes 2009 9;2(5):429–436. 10.1161/CIRCOUTCOMES.108.808592 20031873

[79] Perlis RH, Ganz DA, Avorn J, Schneeweiss S, Glynn RJ, Smoller JW, et al Pharmacogenetic testing in the clinical management of schizophrenia: a decision-analytic model. J Clin Psychopharmacol 2005 Oct;25(5):427–434. 16160617 10.1097/01.jcp.0000177553.59455.24

[80] Perlis RH, Patrick A, Smoller JW, Wang PS. When is pharmacogenetic testing for antidepressant response ready for the clinic? A cost-effectiveness analysis based on data from the STAR*D study. Neuropsychopharmacology 2009 9;34(10):2227–2236. 10.1038/npp.2009.50 19494805 PMC3312011

[81] Priest VL, Begg EJ, Gardiner SJ, Frampton CM, Gearry RB, Barclay ML, et al Pharmacoeconomic analyses of azathioprine, methotrexate and prospective pharmacogenetic testing for the management of inflammatory bowel disease. Pharmacoeconomics 2006;24(8):767–781. 16898847 10.2165/00019053-200624080-00004

[82] Sax PE, Islam R, Walensky RP, Losina E, Weinstein MC, Goldie SJ, et al Should resistance testing be performed for treatment-naive HIV-infected patients? A cost-effectiveness analysis. Clin Infect Dis 2005 11 1;41(9):1316–1323. 16206108 10.1086/496984

[83] Schackman BR, Scott CA, Walensky RP, Losina E, Freedberg KA, Sax PE. The cost-effectiveness of HLA-B*5701 genetic screening to guide initial antiretroviral therapy for HIV. AIDS 2008 10 1;22(15):2025–2033. 10.1097/QAD.0b013e3283103ce6 18784465 PMC2648845

[84] Schalekamp T, Boink GJ, Visser LE, Stricker BH, de Boer A, Klungel OH. CYP2C9 genotyping in acenocoumarol treatment: is it a cost-effective addition to international normalized ratio monitoring? Clin Pharmacol Ther 2006 6;79(6):511–520. 16765138 10.1016/j.clpt.2006.03.008

[85] Sendi P, Gunthard HF, Simcock M, Ledergerber B, Schupbach J, Battegay M, et al Cost-effectiveness of genotypic antiretroviral resistance testing in HIV-infected patients with treatment failure. PLoS One 2007 1 24;2(1):e173 17245449 10.1371/journal.pone.0000173 PMC1769464

[86] Siebert U, Sroczynski G, Aidelsburger P, Rossol S, Wasem J, Manns MP, et al Clinical effectiveness and cost effectiveness of tailoring chronic hepatitis C treatment with peginterferon alpha-2b plus ribavirin to HCV genotype and early viral response: a decision analysis based on German guidelines. Pharmacoeconomics 2009;27(4):341–354. 10.2165/00019053-200927040-00006 19485429

[87] Smith KJ, Monsef BS, Ragni MV. Should female relatives of factor V Leiden carriers be screened prior to oral contraceptive use? A cost-effectiveness analysis. Thromb Haemost 2008 9;100(3):447–452. 18766261

[88] Tavadia SM, Mydlarski PR, Reis MD, Mittmann N, Pinkerton PH, Shear N, et al Screening for azathioprine toxicity: a pharmacoeconomic analysis based on a target case. J Am Acad Dermatol 2000 4;42(4):628–632. 10727309

[89] van den Akker-van Marle ME, Gurwitz D, Detmar SB, Enzing CM, Hopkins MM, Gutierrez de Mesa E, et al Cost-effectiveness of pharmacogenomics in clinical practice: a case study of thiopurine methyltransferase genotyping in acute lymphoblastic leukemia in Europe. Pharmacogenomics 2006 7;7(5):783–792. 16886902 10.2217/14622416.7.5.783

[90] Veenstra DL, Harris J, Gibson RL, Rosenfeld M, Burke W, Watts C. Pharmacogenomic testing to prevent aminoglycoside-induced hearing loss in cystic fibrosis patients: potential impact on clinical, patient, and economic outcomes. Genet Med 2007 10;9(10):695–704. 18073583 10.1097/gim.0b013e318156dd07

[91] Vegter S, Perna A, Hiddema W, Ruggenenti P, Remuzzi G, Navis G, et al Cost-effectiveness of ACE inhibitor therapy to prevent dialysis in nondiabetic nephropathy: influence of the ACE insertion/deletion polymorphism. Pharmacogenet Genomics 2009 9;19(9):695–703. 10.1097/FPC.0b013e3283307ca0 19696696

[92] Weinstein MC, Goldie SJ, Losina E, Cohen CJ, Baxter JD, Zhang H, et al Use of genotypic resistance testing to guide hiv therapy: clinical impact and cost-effectiveness. Ann Intern Med 2001 3 20;134(6):440–450. 11255519 10.7326/0003-4819-134-6-200103200-00008

[93] Welton NJ, Johnstone EC, David SP, Munafo MR. A cost-effectiveness analysis of genetic testing of the DRD2 Taq1A polymorphism to aid treatment choice for smoking cessation. Nicotine Tob Res 2008 1;10(1):231–240. 10.1080/14622200701767761 18188764 PMC2257987

[94] Winter J, Walker A, Shapiro D, Gaffney D, Spooner RJ, Mills PR. Cost-effectiveness of thiopurine methyltransferase genotype screening in patients about to commence azathioprine therapy for treatment of inflammatory bowel disease. Aliment Pharmacol Ther 2004 9 15;20(6):593–599. 15352906 10.1111/j.1365-2036.2004.02124.x

[95] You JH, Chan FW, Wong RS, Cheng G. The potential clinical and economic outcomes of pharmacogenetics-oriented management of warfarin therapy—a decision analysis. Thromb Haemost 2004 9;92(3):590–597. 15351856 10.1160/TH04-03-0161

[96] You JH, Tsui KK, Wong RS, Cheng G. Potential clinical and economic outcomes of CYP2C9 and VKORC1 genotype-guided dosing in patients starting warfarin therapy. Clin Pharmacol Ther 2009 11;86(5):540–547. 10.1038/clpt.2009.104 19571807

[97] Daly TM, Dumaual CM, Miao X, Farmen MW, Njau RK, Fu DJ, et al Multiplex assay for comprehensive genotyping of genes involved in drug metabolism, excretion, and transport. Clin Chem 2007 7;53(7):1222–1230. 17510302 10.1373/clinchem.2007.086348

[98] Antonanzas F, Rodriguez-Ibeas R, Hutter MF, Lorente R, Juarez C, Pinillos M. Genetic testing in the European Union: does economic evaluation matter? Eur J Health Econ 2012 10;13(5):651–661. 10.1007/s10198-011-0319-x 21598012

[99] Frederix GW, Severens JL, Hovels AM, Raaijmakers JA, Schellens JH. The cloudy crystal ball of cost-effectiveness studies. Value Health 2013 Sep-Oct;16(6):1100–1102. 10.1016/j.jval.2013.06.012 24041361

[100] Verhoef TI, Redekop WK, van Schie RM, Bayat S, Daly AK, Geitona M, et al Cost-effectiveness of pharmacogenetics in anticoagulation: international differences in healthcare systems and costs. Pharmacogenomics 2012 9;13(12):1405–1417. 22966889 10.2217/pgs.12.124

[101] NICE. Guide to the methods of technology appraisal 2013. 2013; Available at: https://www.nice.org.uk/article/pmg9/resources/non-guidance-guide-to-the-methods-of-technology-appraisal-2013-pdf. 27905712

[102] Health Care Insurance Board. Dutch pharmacoeconomic guidelines [in Dutch]. 2006; Available at: http://www.zorginstituutnederland.nl/binaries/content/documents/zinl-www/documenten/publicaties/publications-in-english/2006/0604-guidelines-for-pharmacoeconomic-research/0604-guidelines-for-pharmacoeconomic-research/Guidelines+for+pharmacoeconomic+research.pdf.

[103] Goldstein JA. Clinical relevance of genetic polymorphisms in the human CYP2C subfamily. Br J Clin Pharmacol 2001 10;52(4):349–355. 11678778 10.1046/j.0306-5251.2001.01499.x PMC2014584

[104] Lundh A, Sismondo S, Lexchin J, Busuioc OA, Bero L. Industry sponsorship and research outcome. Cochrane Database Syst Rev 2012 12 12;12:MR000033. 23235689 10.1002/14651858.MR000033.pub2

[105] Lexchin J, Bero LA, Djulbegovic B, Clark O. Pharmaceutical industry sponsorship and research outcome and quality: systematic review. BMJ 2003 5 31;326(7400):1167–1170. 12775614 10.1136/bmj.326.7400.1167 PMC156458

[106] Egger M, Juni P, Bartlett C, Holenstein F, Sterne J. How important are comprehensive literature searches and the assessment of trial quality in systematic reviews? Empirical study. Health Technol Assess 2003;7(1):1–76. 12583822

[107] Higashi MK, Veenstra DL. Managed care in the genomics era: assessing the cost effectiveness of genetic tests. Am J Manag Care 2003 7;9(7):493–500. 12866628

