# Supplementary material for: Economic Evaluations of Pharmacogenetic and Pharmacogenomic Screening Tests: A Systematic Review. Second Update of the Literature
Source: PLoS One. 2016 Jan 11;11(1):e0146262. doi: 10.1371/journal.pone.0146262 (PMC4709231; doi:10.1371/journal.pone.0146262)
Supplement: S1 Table — (DOCX) [file pone.0146262.s002.docx]

**S1 Table. Overview of pharmaco-economic PGx studies published between August 2010 and September 2014 analysing the intrinsic value of a PGx test (A), or comparing different treatment strategies involving PGx testing (B).**

**Table A**

| **1^st^ Author** (reference) | **Disease area** | **PGx test** | **PGx test costs** | **Drug** | **Analysis** | **Sensitivity**  **Analyses** | **Time horizon** | **Discounting** | **Perspective** |
| --- | --- | --- | --- | --- | --- | --- | --- | --- | --- |
| **Klang** 2010 **(17)** | ER^+^ LN^-^  ESBC | *21 gene assay* | $3,460 | PGx test+ chemotherapy/no chemotherapy  Vs. chemotherapy/no chemotherapy. | CUA | UV and PB | 30 yrs and lifetime | 3% | Payer’s |
| **Bacchi**  2010 **(18)** | ER^+^ ; ALN ^–^ ESBC | *21 gene assay* | $2,294 | PGx test + chemotherapy/no chemotherapy  *vs. chemotherapy* | CMA | UV | Not mentioned | No discounting | Third party payer’s |
| **Hall**  2012 **(19)** | ER^+^ LN^+^  ESBC | *21 gene assay* | £2,000- £7,000  £2,576 | PGx test + chemotherapy/no chemotherapy  *vs. chemotherapy* | CUA | UV and PB | 30yrs and lifetime | 3.5% | Payer’s |
| **Vanderlaan** 2011 **(20)** | ER^+^ N^+^ -HER2^-^  ESBC | *21 gene assay* | $3,975 | PGx test + chemotherapy/ no chemotherapy *vs. chemotherapy* | CUA | UV | 30yrs | 3% | Payer’s |
| **Verhoef** 2013 **(21)** | AF | *CYP2C9 and VKORC1* | €20- €160  Base case: €40 | Phenprocoumon  *vs.* PGx test + phenprocoumon | CUA | UV, SA and PB | Lifetime | 4% (cost)  1.5% (effects) | Health Care (Payers?) |
| **Dong**  2012 **(22)** | Epilepsy | *HLA-B*1502* | $80-$380  Base case: $270 | Carbamazepine  and phenytoin* | CUA | UV and PB | 30yrs | 3% | Payer’s |
| **Tiamkao**  2013 **(23)** | Neurologi-cal diseases | *HLA-B*1502* | 3,00 Baht | Carbamazepine | CMA | None | Unknown | No discounting | Payer’s |
|  |  |  |  |  |  |  |  |  |  |
| **Shiroiwa**  2010 **(24)** | mCRC | *KRAS* | $220- $1,100  Base case: $220 | Cetuximab | CEA + CUA | UV and PB | 2,5yrs | 3% | Payer’s |
| **Blank**  2011 **(25)** | mCRC | *KRAS* and  *BRAF* | € 394 | Cetuximab | CUA | UV and PB | lifetime | 3% | Payer’s |
| **Behl**  2012 **(26)** | mCRC | *KRAS* and/or *BRAF* | *KRAS*:  $112- $336  Base case: $224  *KRAS* and *BRAF:*  $152- $455  Base case: $303 | Cetuximab | CEA | PB | 1, 2, 5 and 10yrs | 3% | Not reported |
| **Shiffman**  2012 **(27)** | CVD | *LPA* | $100- $200  Base case: $150 | Aspirin | CUA | UV and PB | 10yrs | 3.5% | Payer’s |
| **Donnan**  2011 **(28)** | acute lymphoblastic leukaemia | *TPMT* | $83- $414  Base case: $380 | 6-Mercaptopurine | CEA | UV and PB | 3 months | No discounting | Societal |
| **Schackman**  2013 **(29)** | HIV | *UGT1A1* | $10 or $107  Base case: $107 | Atazanavir  vs. PGx test + atazanavir/darunavir* | CUA | UV and PB | lifetime | 3% | Payer’s |

*Authors assumed equal costs and efficacy for different pharmaceuticals
AF, atrial fibrillation; BRAF, v-Raf murine sarcoma viral oncogene homolog B1; CEA, cost-effectiveness analysis; CMA, cost-minimization analysis; CUA, cost-utility analysis; CVD, cardiovascular disease; CYP, cytochrome P-450; ER, oestrogen receptor; ESBC, early stage breast cancer; HER2, Human Epidermal growth factor Receptor 2; HLA, human leukocyte antigen; KRAS, Kirsten rat sarcoma viral oncogene homolog; LPA, lipoprotein-a; (A)axillary LN, lymph node; mCRC, metastatic colorectal cancer; MV, multivariate; PB, probabilistic; PGx, pharmacogenetic; TPMT, thiopurine S-methyltransferase; UGT, UDP-glucuronosyltransferase; UV, univariate; VKORC, Vitamin K epoxide reductase complex.

**Table B**

| **1^st^ Author** (reference) | **Disease area** | **PGx test** | **PGx test costs** | **Drug** | **Analysis** | **Sensitivity**  **Analyses** | **Time horizon** | **Discounting** | **Perspective** |
| --- | --- | --- | --- | --- | --- | --- | --- | --- | --- |
| **Olgiati**  2012 **(30)** | Depression | *5-HTTLPR* | $100- $300  Base care: $200 | Citalopram and/or bupropion  *vs. PGx test + citalpram and/or bupropion.* | CUA | UV, MV, and PB | Short term | No discounting | Payer’s |
| **Serretti**  2011 **(31)** | Depression | *5-HTTLPR* | $142- $326  Base case: $234 | Citalopram or bupropion  *vs. PGx test + citalopram/ bupropion* | CUA | UV, MV, and PB | 12 weeks | No discounting | Payer’s |
| **Reed** 2011**(32)** | Prostate cancer | 8-14 risk alleles | $200- $600 Base case: $400 | No chemotherapy  *vs. PGx test + finasteride* | CUA | UV and PB | Lifetime | 3% | Payer’s |
| **Djalalov** 2012 **(33)** | Amnesitc cognitive impairment | *APOE ε4* | CAN$150-CAN$1,625  Base case: CAN$325 | Standard of care  *vs. donepezil + PGx test* | CUA | UV and PB | 30yrs | 5% | Societal |
| **Reese**  2012 **(34)** | ACS | *CYP2C19* | $310 | - Clopidogrel   *vs. PGx test + clopidogrel/prasugrel ;*  - Prasugrel  *vs. PGx test+ clopidogrel/prasugrel;* - Generic clopidogrel  *vs. PGx test + generic clopidogrel/ prasugrel;*  - Prasugrel   *vs. generic clopidogrel/prasugrel* | CEA | PB | 15 months | 5% | Payer’s |
| **Sorich**  2013 **(35)** | ACS | *CYP2C19* | AUS$47 | - Clopidogrel  *vs. PGx test + clopidogrel/ticagrelor*  - PGx test + clopidogrel/ticagrelor   *vs. ticagrelor* | CUA | UV and PB | Lifetime | 5% | Healthcare / (Payers?) |
| **Crespin** *.* 2011 **(36)** | ACS | *CYP2C19*  **2* | $100- $300  Base case: $200 | Ticagrelor  *vs. PGx test + clopidogrel* | CUA | UV and PB | 5yrs | 3% | Payer’s |
| **Kazi**  2014 **(37)** | ACS | *CYP2C19*  **2* | $100-$700  Base case: $235 | Clopidogrel  *- vs. prasugrel;*  - *vs ticagrelor*;  - *vs. PGx test + clopidogrel/ ticagrelor  - vs. PGx test+ clopidogrel/ prasugrel* | CUA | UV, SA, and PB | Lifetime | 3% | Societal |
| **Lala**  2013 **(38)** | ACS | *CYP2C19*  **2* | $60-$750  Base case: $500 | PGx test + clopidogrel/ prasugrel  *- vs. clopidogrel*  *- vs. prasugrel* | CUA | UV and PB | 15 months and 10yrs | 3% | Payer’s |
| **Panattoni**  2012 **(39)** | ACS | *CYP2C19*  **2* | NZ$175 | - Clopidogrel  *vs. PGx test + clopidogrel/prasugrel*;  - Prasugrel   *vs. PGx test+ clopidogrel/prasugrel*. | CUA | PB | Lifetime | 3% | Payer’s |
| **Pink**  2013 **(40)** | Nonvalvular AF | *CYP2C9* and *VKORC1* | £20 | Warfarin *vs. PGx test+ warfarin/novel oral anticoagulants.* | CUA | UV and PB | Lifetime | 3.5% | Payer’s |
| **You** 2014 **(41)** | AF | *CYP2C9* and *VKORC1* | $50-$200  Base case: $75 | Warfarin  *vs. PGx test+ warfarin/novel oral anticoagulants.* | CUA | UV, MV and PB | 25yrs | 3% | Payer’s |
| **You**  2012 **(42)** | AF | *CYP2C9* and *VKORC1* | $50-$200  Base case: $72 | PGx test +warfarin *- vs. warfarin; - vs. dabigatran 150mg;*  *- vs. dabigatran mg.* | CUA | UV, MV and PB | Lifetime | 3% | Payer’s |
| **de Lima Lopes**  2012 **(43)** | NSCLC | *EGFR* | SG$190-SG$760  Base case: SG$380 | Standard chemotherapy  *vs. PGx test + gefitinib/standard chemotherapy* | CUA | UV and SA | Lifetime | No discounting | Payer’s |
| **Handorf**  2012 **(44)** | NSCLC | *EGFR* | $122-$365  Base case: $243 | Platinum combination *vs. PGx test + erlotinib*/platinum combination | CUA | UV and MV | 12 months | No discounting | Payer’s |
| **Zhu**  2013 **(45)** | NSCLC | *EGFR* | $381- $635  Base case: $508 | Routine care  *vs. PGx test + gefitinib/routine are.* | CEA and CUA | UV and PB | 10yrs | 3% | Payer’s |
| **Kauf**  2010 **(46)** | HIV | *HLA-B*5701* | $50- $150  Base case: $88 | Abacavir+lamivudine+ efavirenz  *vs.PGx test +abacavir+lamivudine+efavirenz/*tenofovir+emtricitabine | CEA | UV | 60 days and  Lifetime | No discounting | Payer’s |
| **Rattana- vipapong** 2013 **(47)** | Epilepsy and neuropatic pain | *HLA-B *1502* | THB 1,000 | Carbamazepine  *- vs. PGx test + carbamazepine;*  *- vs. alternative (sodium valproate and gabapentin.)* | CUA | PB | Lifetime | 3.5% | Payer’s |
| **Liu**  2012 **(48)** | Chronic hepatitis C | IL-28B | $186-$557  Base case: $371 | Standard therapy (interferon + ribavirin)  *vs. PGx + standard therapy /triple therapy (interferon +ribavirin + protease inhibitor)* | CUA | UV and PB | Lifetime | 3% | Societal |
| **Greeley** 2011 **(49)** | Neonatal diabetes mellitus | *KCNJ11* and  *ABCC8* | $500- $5,000  Base case:  *KCNJ11:* $705 *ABCC8:* $2,110 | Insulin  *vs. PGx test + sulfonylurea* | CUA | UV and PB | 10, 20 and 30yrs | 3% | Societal |
| **Parthan** 2013**(50)** | ACS | *KIF6* | $100 | Pravastatin  *vs. PGx test + pravastatin/atorvastatin* | CUA | UV and PB | Lifetime | 3% | Payer’s |
| **Vijayara-ghavan**  2012 **(51)** | mCRC | *KRAS* | $243 | - Panitumumab   *vs. PGx test +*  *panitumumab/other chemo;*  - Cetuximab   *vs. PGx test + cetuximab/*  *other chemotherapy; -* Combination therapy (cetuximab + irinotecan ) *vs. PGx test + combination therapy /irinotecan.* | CEA | UV | Lifetime | No discounting | Payer’s |
| **Hagaman**  2010 **(53)** | idiopathic pulmonary fibrosis | *TPMT* | $300 | Azathioprine *vs. PGx test+ azathioprine/alternative* | CUA | UV and MV | lifetime | No discounting | Payer’s |
| **Thompson**. 2014 **(52)** | Autoimmune disease | *TPMT* | £20 | Azathioperine  *vs. PGx test+ azathioprine/alternative* | CUA | UV | 4 months | No discounting | Health service |
| **Pichereau** 2010 **(54)** | mCRC | *UGT1A1 *28* | €71 | FOLFIRI  *vs. PGx test + FOLFIRI/ FOLFIRI+ CSF* | CEA | UV and PB | 2 weeks | No discounting | Hospital’s |

ABCC, ATP-binding cassette transporter syb family C; ACS, acute coronary syndrome; AF, atrial fibrillation; APOE, apolipoprotein-E; CEA, cost-effectiveness analysis; CSF, Colony Stimulating Factor ;CUA, cost-utility analysis; CYP, cytochrome P-450; EGFR, epidermal growth factor receptor; FOLFIRI , Folinic acid, fluorouracil, irinotecan; HLA, human leukocyte antigen; HTTLPR, serotonin-transporter-linked polymorphic region; IL, interleukine; KCNJ, Potassium inwardly-rectifying channel, subfamily J; KIF, kinase family member; KRAS, Kirsten rat sarcoma viral oncogene homolog; LPA, lipoprotein-a; mCRC, metastatic colorectal cancer; MV, multivariate; NSCLC, non-small cell lung cancer; PB, probabilistic; PGx, pharmacogenetic; SA, scenarios analyses; THB, Thai Baht; TPMT, thiopurine S-methyltransferase; UGT, UDP-glucuronosyltransferase; UV, univariate; VKORC, Vitamin K epoxide reductase complex.
