# Supplementary material for: Economic Evaluations of Pharmacogenetic and Pharmacogenomic Screening Tests: A Systematic Review. Second Update of the Literature
Source: PLoS One. 2016 Jan 11;11(1):e0146262. doi: 10.1371/journal.pone.0146262 (PMC4709231; doi:10.1371/journal.pone.0146262)
Supplement: S2 Table — (DOCX) [file pone.0146262.s003.docx]

**Supplementary file 2:** Main limitations which were discussed in the papers published between August 2010 and September 2014 .

| **Study** | **Discussion of limitations** |
| --- | --- |
| Bacchi  *et al.,* 2010 | - The hypothetical nature inherent to economic models - Not all direct and indirect costs were taken into account - The effectiveness of therapy based on the risk prediction of the assay was not considered - There was variability in the definition and incidence of febrile neutropenia - It was not possible to include Brazilian estimates for stage distribution upon diagnosis of breast cancer |
| Behl  *et al.* 2012 | - No progression-free survival outcomes for the different strategies was provided - Limitations in data for differences among the treatment options in quality of life, and was therefore not incorporated - Gain in overall survival may not have been seen if the health impact of treatment were expressed in quality-adjusted survival - Differences in quality of life among patients receiving alternate treatments have not been quantified in a way that allows quality adjusted survival to be modelled - It was not taken in account that patients could discontinue the treatment due to adverse events - The analysis assumes that all differences in survival are due to a lack of response to cetuximab. Though mutations may independently predict prognosis, not the kind of treatment - The estimated savings might be overestimated if any part of the survival difference is due to mutations, and independent of the therapy |
| Blank  *et al.*2011 | - Lack of clinical data of a clearly define patient population from Switzerland. Hence, clinical and utility data originated from few studies conducted outside Switzerland - Uncertainty in trial data and potentially limited transferability to routine clinical practice populations - The quality of life and utility data allowed to differentiate on the basis of treatment, but not of mutation status - *BRAF* mutation seemed to have no impact on response to the antibody, suggesting that *BRAF* mutation may not have the same predictive value in first-line and chemorefractory tumours. |
| Crespin  *et al.*2011 | - The ultimate effectiveness of antiplatelet medication likely cannot be determined, because the short duration of the clinical trials - The estimates might be biased if the effectiveness of ticagrelor differs after 12 months of therapy - Results may not apply to subpopulations extracted from clinical trials - The costs for adverse event might vary between subpopulations due to events that significantly differ - Assumptions that MI and death were independent, which is likely unrealistic - Results cannot be used to determine universal cost-effectiveness relative to other viable treatment options for secondary prevention after an acute coronary syndrome |
| de Lima Lopes  *et al.* 2012 | - Assumptions were made about length and time spent in treatment by extrapolating from clinical data, and assuming that the time spent in first- or second-line treatment with chemotherapy of gefitinib was equivalent to account for similar overall survival - Cost data is obtained from 3 Singapore oncology centres, and might not be representative for other countries and/or hospitals - Several assumptions about quality of life values were made, hence the estimated ICERs might be biased - Lack of data to capture robust individual level variation in treatment responses - The standard of care for first line treatment has shift to include pemetrexed, bevacizumab and cetuximab in first-line treatment and to include pemetrexed as potential maintenance option after initial chemotherapy - The model specifically compares a standard practice, which includes no EGFR mutation testing, first-line treatment with chemotherapy and second-line treatment with gefitinib, to EGFR testing and guided therapy based on the results of the test. - In the base case it was assumed that gefitinib did not benefit patients without activating mutations, even beyond first line treatment |
| Djalalov  *et al.*2012 | - There is limited evidence on the effectiveness of donepezil treatment in delaying progression from AMCI to AD among APOE e4 carriers. - Surveillance costs were based on results from a study that used older patients, and was conducted in a different healthcare system - They did not used some data from a study, because that data might have had selection biases that limited the generalisation of the results - Adequate and widely accepted criteria for diagnosing AMCI are unavailable, but incorrect diagnoses not incorporated in the study. Incorrect diagnosing might increase the ICER - They did not included patients that were not seen and diagnosed with AMCI before the development of AD |
| Dong  *et al.*2012 | - The study assumes that health-related QoL is restored to perfect health. However, the QALY gains might be overestimated if the health related QoL is in reality lower due to an imperfect response to drugs, epilepsy recurrence or other health problems - Treatment decisions are in reality influenced by many factors and may substantially deviate from the initial assumptions - The model also includes several additional assumptions and simplifications: - Some patient subpopulations excluded - Treatment rules simplified for who receives the treatment - The model assumes that VPA, CBZ, and PHT have similar efficacy. - Effectiveness data from a study using Caucasians, ethnic differences cannot be ruled out - They assumed that genotyping results are immediately accessible, though this may not hold universally |
| Donnan  *et al.* 2011 | - The rarity of the disease limits amount and type of evidence available, therefore some values based on expert opinion - No QALY analysis possible, due to lack of literature on utility scores for children with ALL - There was some uncertainty in the values used for the unit prices of TPMT genotype and enzymatic tests |
| Greeley  *et al.*2011 | - In this study it was assumed that the test was 100% sensitive and specific, although there was not accounted for factors that might diminish the effective clinical sensitivity and specificity - Lack of data, therefore some of the model’s assumptions for selected complications were derived from studies performed on patients with diabetes type 2 - Due to a lack of quality-of-life data in neonatal diabetes, it was assumed that patients would experience a utility gain of 0.1 based on survey data from diabetes type 2 subjects - Due to insufficient longitudinal data, it was assumed that patients with treatable genetic defects would remain responsive to the treatment over 30years - The model did not account for the therapy’s potential to improve neurodevelopmental outcomes |
| Hagaman  *et al.*2010 | - There were no published trials that specifically explored their issue; therefore they had to draw data from numerous sources. - The study assumed that the low-dose treatment with reduced TPMT activity is the same as for full-dose therapy in patients with normal TPMT activity - The study lacked data describing the incidence of BMT in patients with intermediate TPMT activity - The breakdown of TPMT activity in patients with leukopenia has not been clearly elucidated - The authors assumed that conservative therapy was equal in efficacy to the placebo arm in another study, in which the patients received azathioprine and steroids with a NAC placebo. This assumption likely overestimates the marginal cost-effectiveness of therapy with azathioprine, NAC and steroids. |
| Hall  *et al.* 2012 | - The authors conducted an analysis of the immediate budget impact to illustrate the financial implications during the chemotherapy period only - The budget impact results do not characterise the strength evidence, and do not consider long term costs (for cancer recurrence and treatment toxicity, relative life expectance and quality of life) - Only the Oncotype DX was considered, and not the relative value and alternative tests - It is important to recognize that any model is a simplification of reality, and the model presented here is no exception. - It is credible that taking into account the transfer­ability of data from a US trial into a UK setting would generate additional uncertainty and even introduce bias into the results. - The use of decision aids such as Adjuvant Online! was not specifically incorporated into the model - The possibility that the price of the test might change with the introduction of alternatives was not considered - Estimates of long-term costs and quality of life (e.g., associated with cancer recurrence or cardiac toxicity) are derived from higher quality evidence than the short-term data sources (e.g., costs of toxicity) in this analysis because they rely on previously published dedicated cost and quality of life studies. They are, however, subject to assumptions of data transferability to our patient population and require confir­mation in a formal study with long-term follow-up. - This analysis presents results only for the average patient aged 60 years |
| Handorf  *et al.* 2012 | - The authors were not able to model the full course of lifetime therapy, which often included second- and third-line treatment. The inputs for such a model were not available - A model-based approach was applied, rather than a randomised trial. Therefore, the conclusions depend on the validity of the assumptions that were used to develop the model - The authors were not able to gather costs and effects for claims and registry data - The current analyses were subject to modifications in costs (reduction of costs in the future makes the strategies more favourable) |
| Kauf  *et al.*2010 | - This analysis relied on several assumptions specific to the development of the ADVANCE model as described in the text and the supplemental data. - Care should be taken in the interpretation of the results in which screening dominated the alternative therapy, because differences in effectiveness very small in the dominant screening compared with alternative strategy - The results only apply to those patients for whom abacavir and tenofovir are considered appropriate treatment alternatives - This analysis does not consider all the possible benefits of screening |
| Kazi  *et al.* 2014 | - “Estimated differences in outcomes between various *CYP2C19* genotypes were largely based on post hoc analyses of randomized trials” - The efficacy and safety of prasugrel and ticagrelor were based on only one large, randomized clinical trial - The indirect comparison of ticagrelor with prasugrel was limited due to structural differences in the design and execution of the used clinical trials - It was assumed that the clinical outcomes from the PLATO trial can be translated to U.S. patients on low dose aspirin therapy, however this has yet to be investigated - In order to define the actual relative cost-effectiveness, the long-term effects of newer antiplatelet agents on mortality rate has to be determined |
| Klang  *et al.* 2010 | - The clinical data on which the analyses were based were derived from a non-randomly selected sample of patients - Utilities were derived from English speaking literature, thus may not fully characterise the preferences of patients in Israel - Validation of the essay was based on clinical trials conducted in the US - Due to limited data, the authors omitted some potential long-term implications of breast cancer and its treatments such as the risk of local recurrence and risk of second primary tumours associated with chemotherapy - The authors examined the effect of the test from the payer’s perspective, hence indirect costs were not included |
| Lala  *et al.*2013 | - Healthcare delivering costs were not available at the individual patient level - The authors only accounted for the impact of testing for CYP2C19*2 allele was taken in account, and the cost-effectiveness of genetic testing for other mutations was not taken in account - This analysis did not address the economics of platelet reactivity testing - For patients with a prior history of TIA or stroke, body weight <60 or age >75 prasugrel may not be considered optimal therapy - It was assumed that there was a constant magnitude of benefit and a constant rate of adverse events - The authors assumed that the relative risk of death in our population, compared with the general US population, remained fixed over time. - There studied that found differences in bleeding events between CYP2C19*2 carriers and wild type - Although genetic testing was shown to be cost-saving   compared with treating all ACS patients undergoing PCI  empirically with prasugrel or clopidogrel, the absolute health and cost differences were small |
| Liu  *et al.* 2012 | - Because of a lack of evidence, strategies involving retreatment with triple therapy after initial failure was not taken into account - They did not included the effect of reductions in HCV transmission due to successful treatment - The results are limited to mono-infected individuals |
| Olgiati  *et al.* 2012 | - It assumes that 5-HTTLPR variants have the same distribution and effect size in all European countries. - The influence of 5-HTTLPR on SSRI response is documented in randomized trials, but biased by various limitations - The issue of the transferability of the results was implicitly addressed by considering several regions at different income levels - According the authors it is arguable that reduction in health expenditures for approximately 4% of new responders under pharmacogenetic treatment cannot offset incremental costs for genetic test |
| Panattoni  *et al.* 2012 | - The findings are based on models of outcomes rather than a randomised trial - Whether the shorter duration of clopidogrel therapy contributed to the higher events rates was not clear - The definitions and classification of adverse events differ between New Zealand hospital DRG data and the TRITON-TIMI 38 clinical trial - The ethnicity data has limitations, because many patients had heritage from more than 1 ethnical group |
| Parthan *et al.* 2013 | - The analysis was based on a post hoc substudy, which was not designed to investigate the cost-effectiveness of *KIF6* testing - It was assumed that the patients with a major cardiovascular event remain in this state until death, although they have a higher risk of a second event - Cardiovascular event rates were extrapolated because data was only available for 2 years - The estimates of differential statin benefit may have been overestimated, because the association of *KIF6* with the differential reduction of CHD event rates from statin therapy may be limited to high-dose atorvastatin and standard dose pravastatin - There was no data on event rates for non-adherent patients by *KIF6* carrier status, therefore the 4-month event rate for patients in the placebo arm was used as a proxy - The authors did not accounted for a possible relationship between multiple events and risk of death, therefore the estimation of life expectancy may not be accurate - The cost of secondary events was estimate to be the same as the costs of a primary event - “The acute costs of UA requiring hospitalization is represented in the model as a 36-month cost in light of the absence of data on UA as an event secondary to other cardiovascular events.” |
| Pichereau  *et al.* 2010 | - The authors did not opt for a full economic model in the analysis as the primary intention was to evaluate whether or not UGT1A1 genotype testing would be an efficient use of additional resources from the hospital. - Only few studies were used to build our model as most of the available data were not associated with complete information of polymorphisms prevalence or FN incidence - The authors assumed 100% efficacy of CSF therapy, thus inflating the estimates of neutropenic events avoided, if efficacy is in fact lower. - The costs of CSF were not included because this drug was not provided by the hospital - Indirect and intangible costs were excluded - Irinotecan toxicity might also be affected by other polymorphisms, though this was not taken in account for this study |
| Pink  *et al.* 2013 | - The adjusted indirect comparison is necessary to include all possible treatment options. However, this may introduce bias through differences in trial design, a lack of access to individual patient data and the need to extrapolate the available data from trial to lifetime horizons. - The results were obtained from a number of data sources are difficult to validate externally - Both the PKPD and the economic models are parameter extensive, increasing the probability that some of the values used are inaccurate - Each of the three stages of the methodology introduces uncertainties |
| Rattanavipapong  *et al.* 2013 | - Due to the rarity of SJS/TEN cases, only a small number of patients was included, representing both cost and utility limitations - There is no surveillance system to quantify the prevalence of CBZ-induced SJS/TEN in the Thai population - This study employed data from only one study, which was conducted in a medical school in Thailand |
| Reed  *et al.* 2011 | - The impact of finasteride outside clinical trial settings is unclear. In the PCPT active surveillance for prostate cancer continued throughout the trial, so it is conceivable that the effect of finasteride could be attenuated among patients followed in routine practice - It is also unclear whether the treatment effects measured in the PCPT apply equally to patients at higher risk for prostate cancer, whether treatment effects decrease over time and whether reductions in the prevalence of prostate cancer lead to decreases in prostate cancer specific mortality. - The is no established threshold in the US for determining whether an intervention is cost-effective - Efficiency gains were limited from a cost-effectiveness perspective |
| Reese  *et al.* 2012 | - The authors included both IMs and PMs for prasugrel in the genotype-guided treatment arm, although a boxed warning in the clopidogrel label only includes PMs. However, the majority of the published data indicates that both IMs and PMs are at increased risk. - The probabilities used in the base-case models were obtained from one randomised trial and substudies of that trial. This concerns age and ethnicity - Substudy analyses could have introduced biases - In this study the treatment lasted 15 months, wheras the treatment guidelines recommend antiplatelet therapy for at least one year - The cost of genetic tests will probably influence the use. |
| Schackman  *et al.* 2012 | - The results from a retrospective analysis were used, though they may not be generalizable to the US or other populations - The authors assumed in the base case that atazanavir and darunavir hat equivalent efficacy and costs - The assumption that clinicians or patients might prefer to initiate atazanavir was not captured in the base case QALYs or costs - The range of QoL effects of hyper bilirubinaemia that was considered may not capture the full spectrum of clinical situations - The authors did not consider the potential future benefit of *UGT1A1* testing to inform prescribing of other non-HIV drugs |
| Serretti  *et al.* 2011 | - There was no experimental data about effective gain in antidepressant response due to pharmacogenetic approach. - The assumptions that patients with s/s genotype treated with citalopram would have the greatest benefit from genetic testing would, due to the remission rates, imply that virtually all the antidepressant response in the 5-HTTLPR s/s genotype group was due to a placebo response and none to the SSRI antidepressant - At best estimate 50-60% of change in response outcome might be due to a true pharmacologic effect. - This study focused on antidepressant response and side effects. However the 5-HTTLPR polymorphism has also been found to moderate depressive response to environmental stress - A long follow-up would have been methodologically correct, but constituted a serious hurdle to build a realistic model. Firstly, because only a minority of patients do not drop out from treatment after a few months. Secondly, even in those who remain in treatment, adherence is seldom at optimal level, and this may have negative consequences for effectiveness and cost. For a long-term assessment of major depressive disorder it is necessary to estimate recurrence rate, This is not an easy task, because follow-up studies of depression are characterized by marked differences in terms of designs, outcome definitions and crude measures of pharmacotherapy - The influence of 5-HTTLPR on SSRI response is documented in randomised trials, but biased by various limitations - Less clear evidence came from naturalistic studies - The real effect is not yet established - The authors posted that sensitivity to 5-HTTLPR variants was equivalent for all SSRIs, though recent studies found subtle differences - The model did not account for recent discoveries that changed the structure and function of the 5-HTTLPR polymorphism. - In order to simplify the association between 5-HTTLPR variants and antidepressant response, second-order interactions with gender and life-events were not featured - Information was missing regarding costs to caregiver or family members and psychotherapy - The used typical starting doses for SSRI treatment might be suboptimal and interfere with the assessment of clinical response - The impact of antidepressant treatment on suicidal risk was not featured - The results are only provisional because key assumptions regarding gain in antidepressant response and reduction in side-effect burden are speculative and not supported by empirical findings |
| Shiffman  *et al.* 2012 | - The authors were unable to measure or find a published estimate of the risk of stroke events associated with the 2 variants of the *LPA* gene in women. Therefore, they assumed that the increased stroke risk in women was the same as the increased MI risk estimated in men - It was assumed that frequency of the *LPA* risk alleles is unchanged at different levels of the FRS. - There was no source documenting the timing of GI bleeding events that occur after initiation of aspirin therapy, therefore it was assumed that GI bleeding events would occur in the first year after initiation of therapy - There were no reports of an association between these *LPA* variants and the risk of CVD in populations of non-European ancestry |
| Shiroiwa  *et al.* 2010 | - The authors estimated the BSC costs, which were based on the fee-for-service costs of daily opioid use although some patients do not use them, or less frequently - There was not data available for Japanese utility scores, so Canadian scores were extrapolated |
| Sorich  *et al.* 2013 | - Cost between health systems are different and result may not apply to other healthcare systems - No comparison with prasugrel or genotype guided dosing including alternative genotypes like Ultra Rapid metabolizers. - It was assumed that clopidogrel or ticagrelor were used for 12 months after ACS and then stopped. |
| Thompson  *et al.* 2014 | - The study’s sample may not be generalizable to all potential patients who will be offered *TPMT* genotyping in clinical practice. - The study time horizon should have been longer to capture any potential long-term costs and benefits - The perspective of the study might be a limitation, because it does not take into account the costs beyond health care resource use |
| Tiamkao  *et al.* 2013 | - This study was based on reviews of literature, so the details could be slightly different and could result in discrepancies - Costs of treatment of other complications after discharging the patient, re-admission or out-patient follow up were not included |
| Vanderlaan  *et al.* 2011 | - The estimate of the net reduction in chemotherapy associated with assay testing was based on a single published study for the general N+(1-3)/ER+ population and on a separate analysis for the >65-year age group - The model used an age distribution representative for the US population estimating the incidence of breast cancer, though the model was applied to a potentially younger managed care population - A payer’s perspective was applied, and indirect costs were excluded - Clinical trial data was used to estimate costs of treatment, hospitalisation and medication associated with adverse events, yet the costs are often higher in a non-controlled setting - The authors modelled change in chemotherapy use only among those with low recurrent score results in order to provide a conservative estimate of the benefits gained from the assay’s use in this population - The plan costs of chemotherapy drugs and supportive care may have been overestimated - Unpublished survey data was used to estimate practice patterns of chemotherapy-related supportive care - It was estimated that 90% of the cancers would be non-HER2 overexpressing, although this might be uncertain - The estimated 2.7% excess risk among patients receiving chemotherapy may be an overestimate, because it was based on data that included radiotherapy and hormonal treatment - The model did not address the use of the assay in the population with the current greatest use - The model assumed that cancer and all-cause mortality were estimated based on national statistics but did not incorporate one additional benefit of the oncotype DX assay (if the recurrent score is known, patients’ risk of cancer-related death is stratified into 3 categories) - There is great variability in the value that patients attribute to chemotherapy treatment |
| Verhoef  *et al. 2013* | - Lack of data concerning the effectiveness of genotyping in phenprocoumon patients from clinical trials. - Cost of genetic test - Surrogate end point (INR) - Possible correlation between parameters which were independently varied in the probabilistic sensitivity analysis |
| Vijayaraghavan  *et al.* 2012 | - The analysis was restricted to the use of EGFR inhibitors in second and subsequent lines of therapies - It was not taken in account that recent findings indicated that use of cetuximab in chemotherapy-refractory colorectal cancer patients is associated with longer overall survival compared to patients with other KRAS-mutated tumours - Due to a lack of data on utilities for patients with mCRC receiving therapy, no quality of life adjustments were incorporated in the model - There is limited data on the precise sensitivity and specificity of the KRAS test, therefore the authors estimated a 95% sensitivity and 100% specificity |
| You  *et al.* 2012 | - The model projected life-long events based on key factors derived from a 2 years clinical trial - The cost items were limited to the resources of anticoagulation therapy and related complications |
| You JH,  2014 | - Long-term events were projected by using short-term clinical trial data - Monitoring of new data regarding NOACs is required to update the decision model - The cost items were limited to resources of anticoagulation therapy and related complications - A TTR of 60% was assumed, which might not be applicable to clinics with higher TTR - In the CoumaGen-II trial patients with various warfarin indications were mixed, which could result in uncertainty in the estimation of genotype-guided dosing effectiveness in AF patients in this study. |
| Zhu  *et al.* 2013 | - The current analysis did not evaluate the cost-effectiveness of gefitinib maintenance treatment for the whole cohort without EGFR genotyping because this data was not available - The present model did not include other EGFR-targeted agents used as maintenance treatments, such as erlotinib, to assess the incremental cost-effectiveness in comparison with gefitinib because no head-to-head trial data are currently available. - A budget impact analysis for the addition of gefitinib maintenance treatment on society was not conducted - The current analysis incorporated PSF and OS data after cancer progression from different trials. - Some model inputs were obtained from literature published abroad due to a lack of Chinese clinical data - The sensitivity and specificity of different genotyping facilities was not accounted - In order to simplify the evaluation, other adjuvant therapies were excluded |
